# Supplementary figures and images for: Obscurin Rho GEF domains are phosphorylated by MST-family kinases but do not exhibit nucleotide exchange factor activity towards Rho GTPases in vitro
Source: PLoS One. 2023 Apr 20;18(4):e0284453. doi: 10.1371/journal.pone.0284453 (PMC10118190; doi:10.1371/journal.pone.0284453)

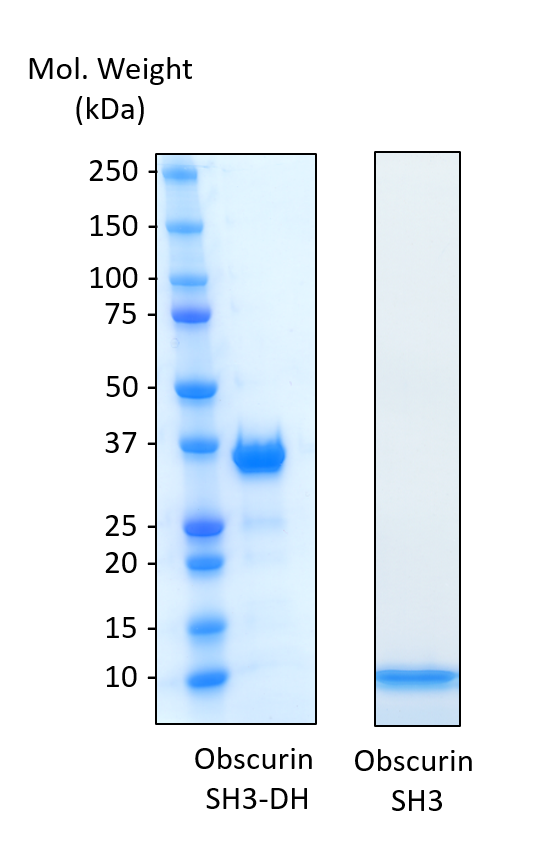

Supplement: S1 Fig — SDS-PAGE analysis of obscurin SH3-DH (amino acids 5601–5899) and SH3 domains (amino acids 5601–5667) after affinity purification and size-exclusion chromatography. (PNG) [file pone.0284453.s001.png]

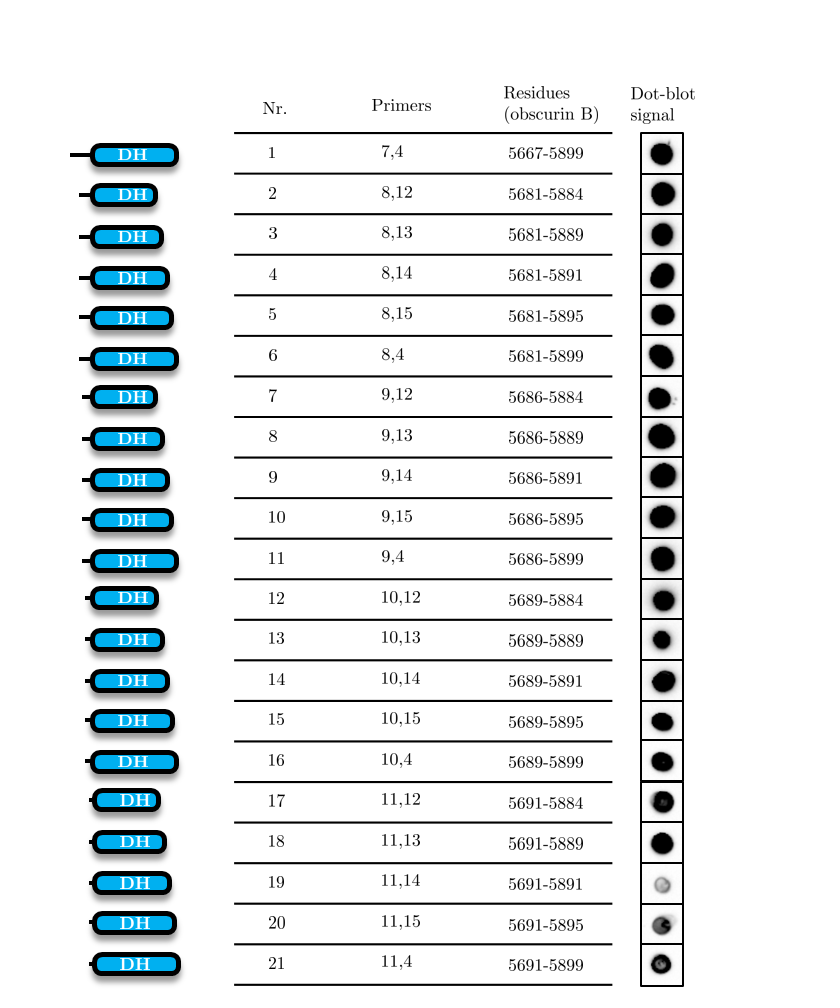

Supplement: S2 Fig — Construct design, primers used and dot-blot analysis of the eluted fraction after affinity purification of the human obscurin DH, DH-PH and PH domains. (PNG) [file pone.0284453.s002.png]

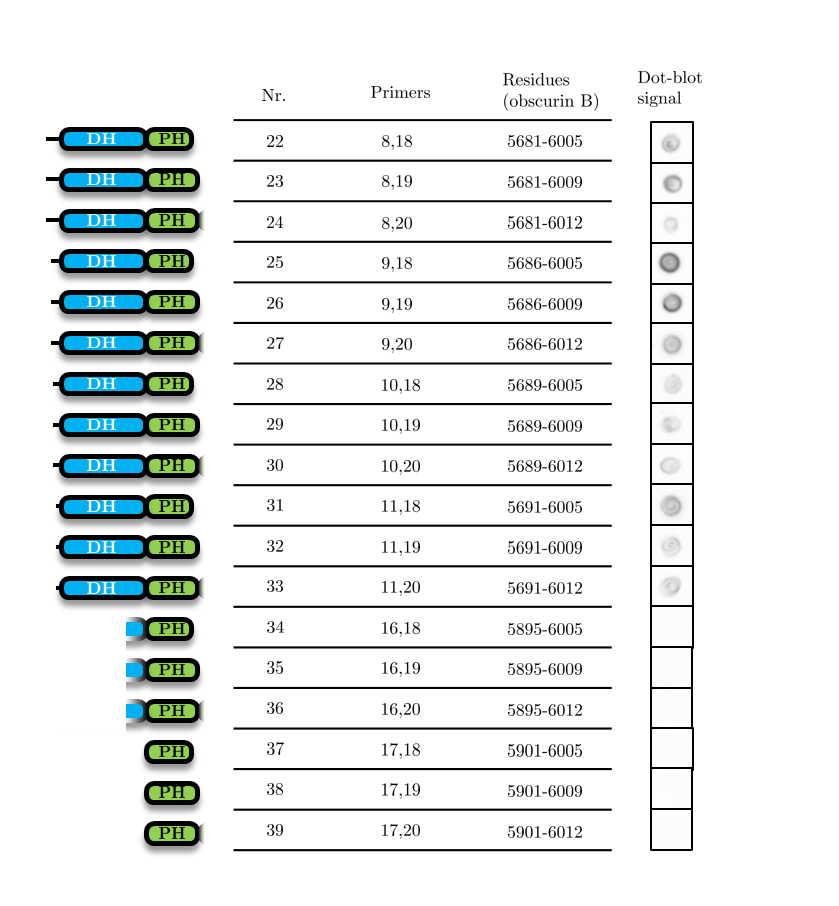

Supplement: S3 Fig — Construct design, primers used and dot-blot analysis of the eluted fraction after affinity purification of the human obscurin DH, DH-PH and PH domains (continued). (PNG) [file pone.0284453.s003.png]

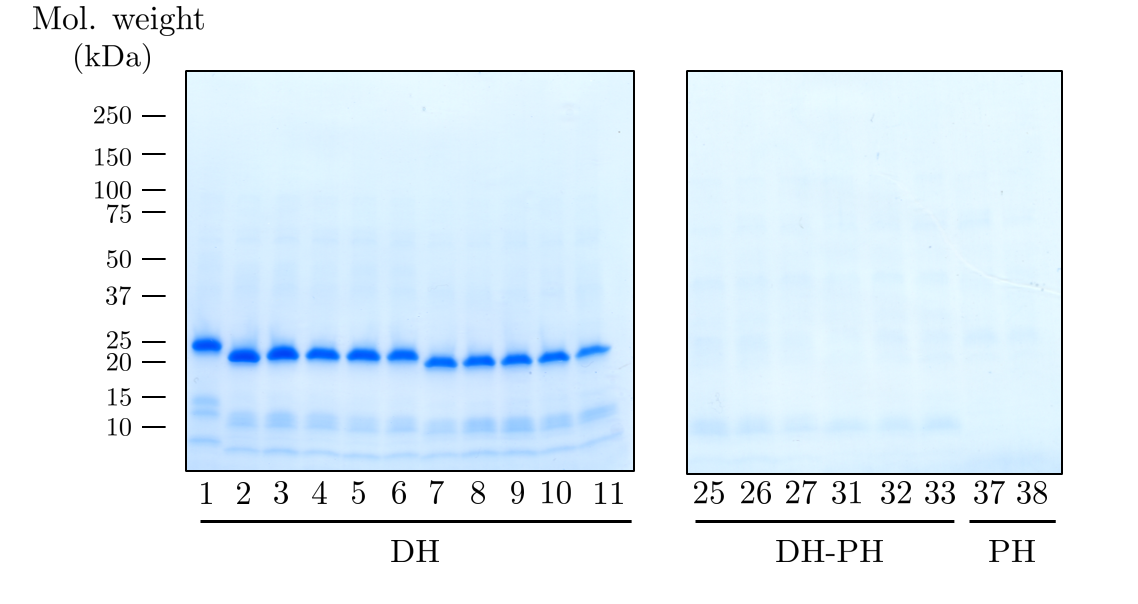

Supplement: S4 Fig — SDS-PAGE analysis of selected fragments after affinity purification. The same samples shown in the dot-blots of S1 and S2 Figs were used for SDS-PAGE. (PNG) [file pone.0284453.s004.png]

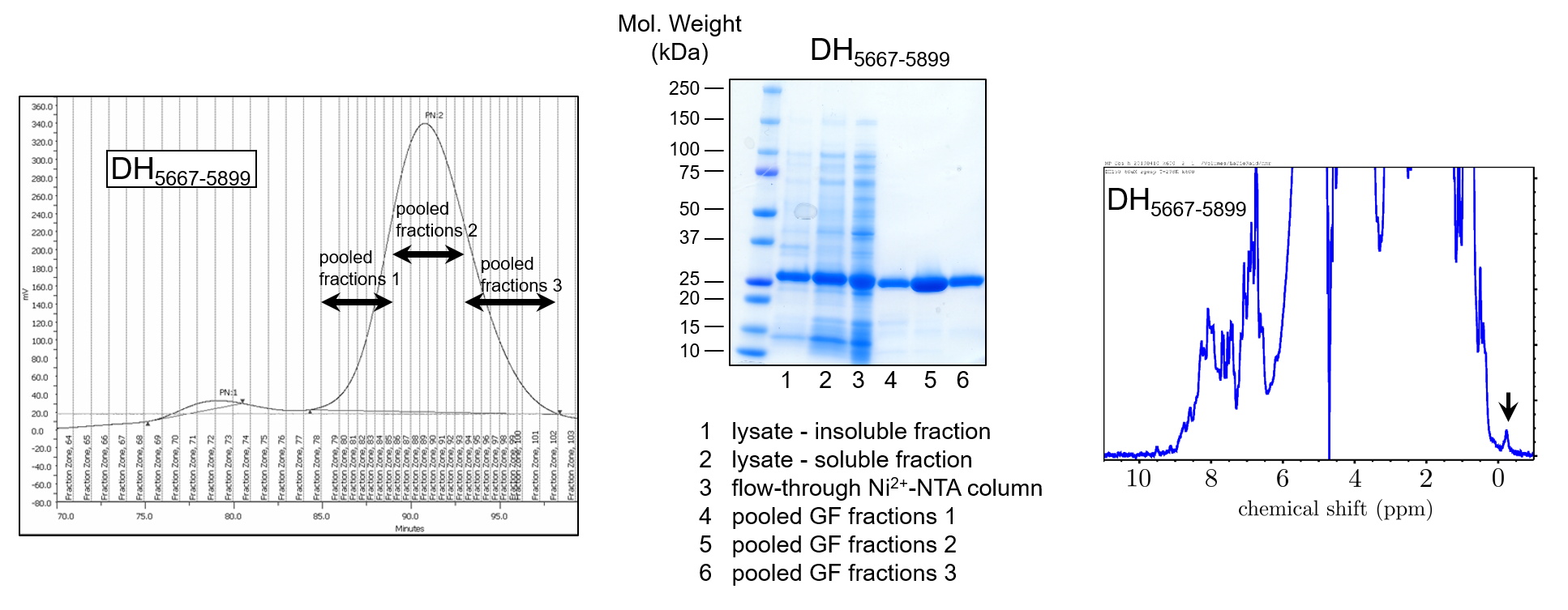

Supplement: S5 Fig — Left, gel-filtration elution profile of DH domain fragment comprising residues 5667–5899. Middle, SDS-PAGE analysis of samples at different steps of purification process of DH5667-5899 fragment. Right, 1D-NMR analysis of purified DH5667-5899 fragment. NMR spectrum exhibits wide peak dispersal and peaks below 0 ppm (black arrow), indicating that the protein is folded. (PNG) [file pone.0284453.s005.png]

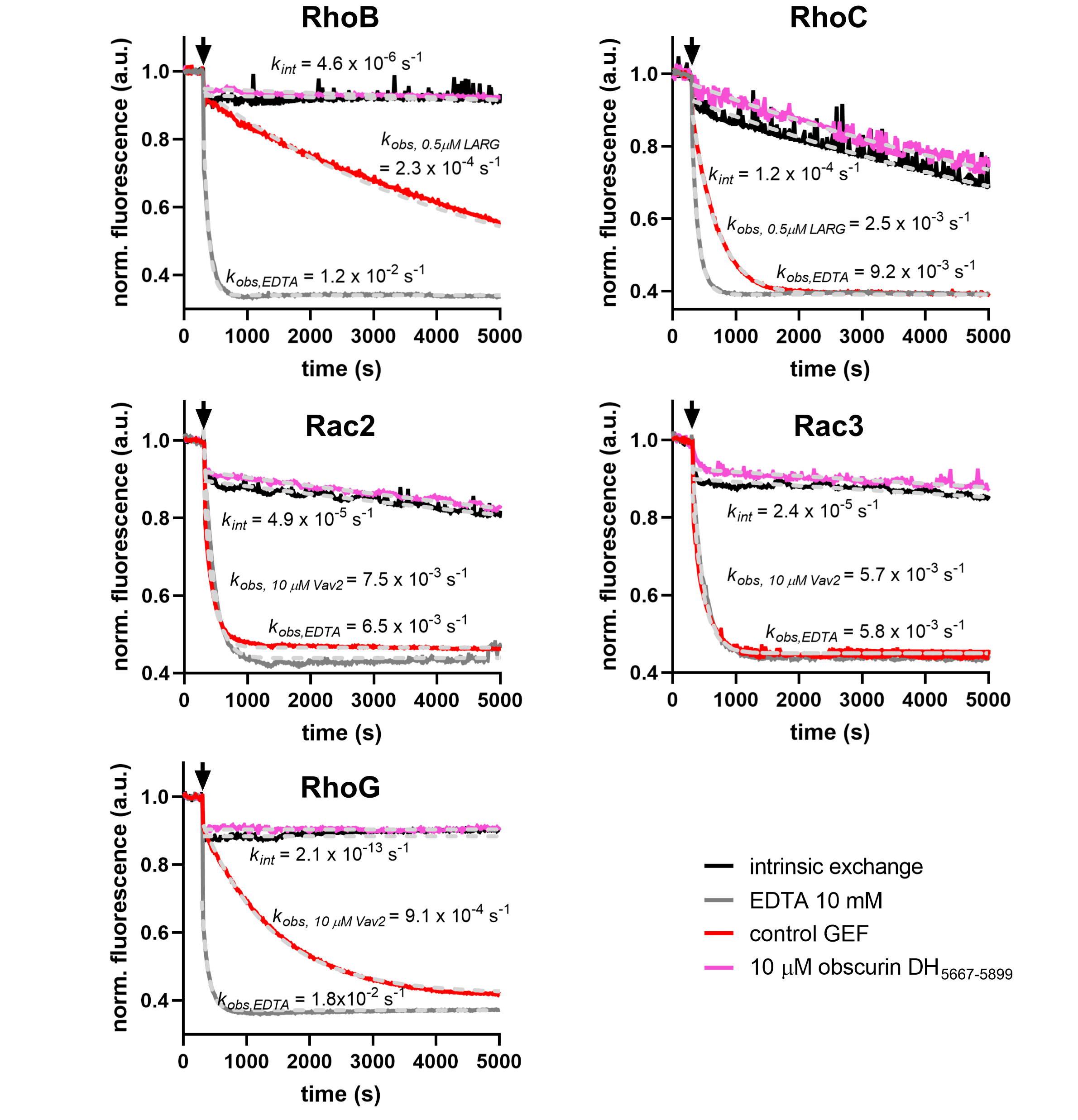

Supplement: S6 Fig — Black arrows indicate addition of buffer/GEF/EDTA. Data represent mean of n = 2–3 experiments. (PNG) [file pone.0284453.s006.png]

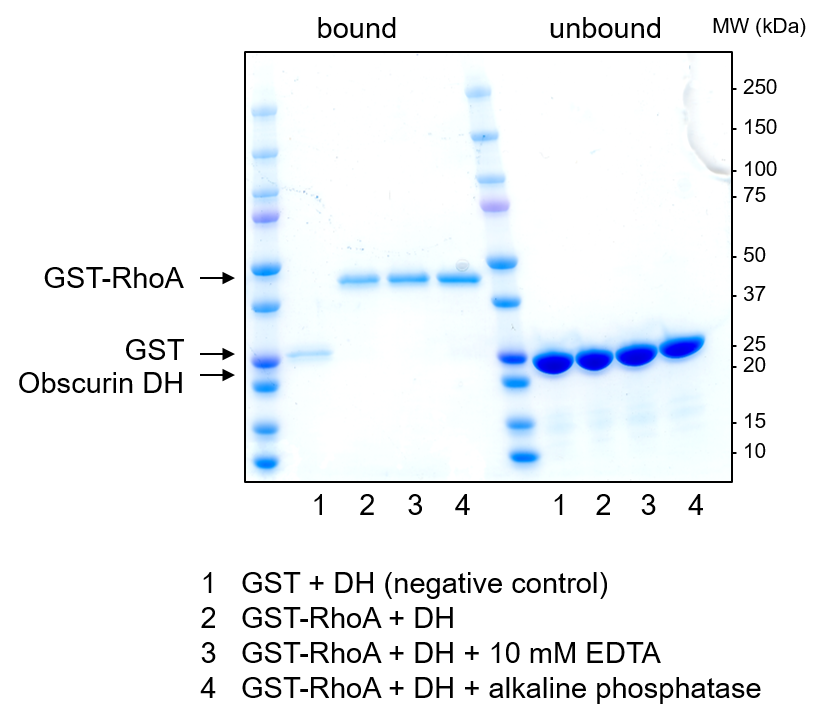

Supplement: S7 Fig — Addition of EDTA or alkaline phosphatase lead to a nucleotide free state of RhoA. (PNG) [file pone.0284453.s007.png]

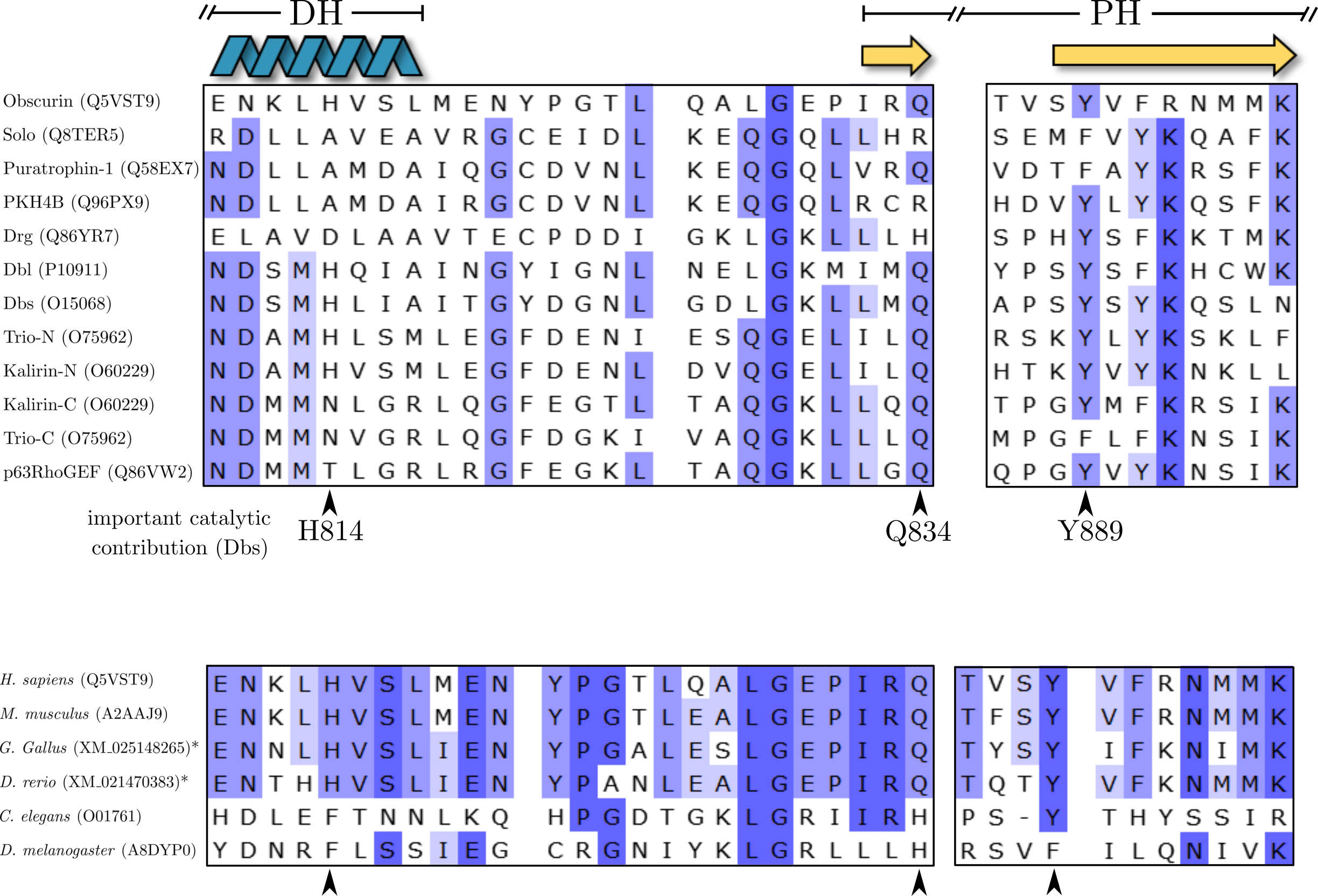

Supplement: S8 Fig — Black arrowheads indicate important catalytic contribution (numbering refers to Dbs sequence). Conserved residues are highlighted in blue with colorintensity highlighting the degree of conservation. Top panel shows comparison of human Trio-subfamily GEFs. Bottom panel shows obscurin DH-PH domain interface across different species. The unique Uniprot-identifier for each protein is given in brackets. When the protein was not available on Uniprot (*), the NCBI-Reference sequence is given instead. (PNG) [file pone.0284453.s008.png]

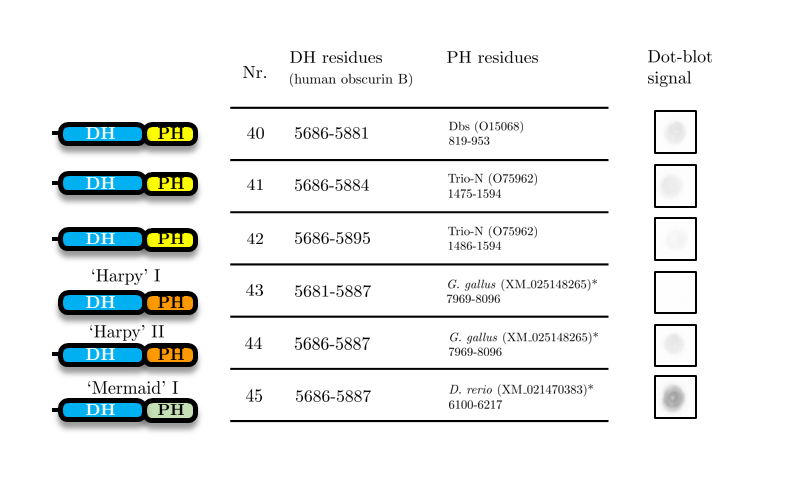

Supplement: S9 Fig — The unique Uniprot-identifier for each protein is given in brackets. When the protein was not available on Uniprot (*), the NCBI-Reference sequence is given instead. (PNG) [file pone.0284453.s009.png]

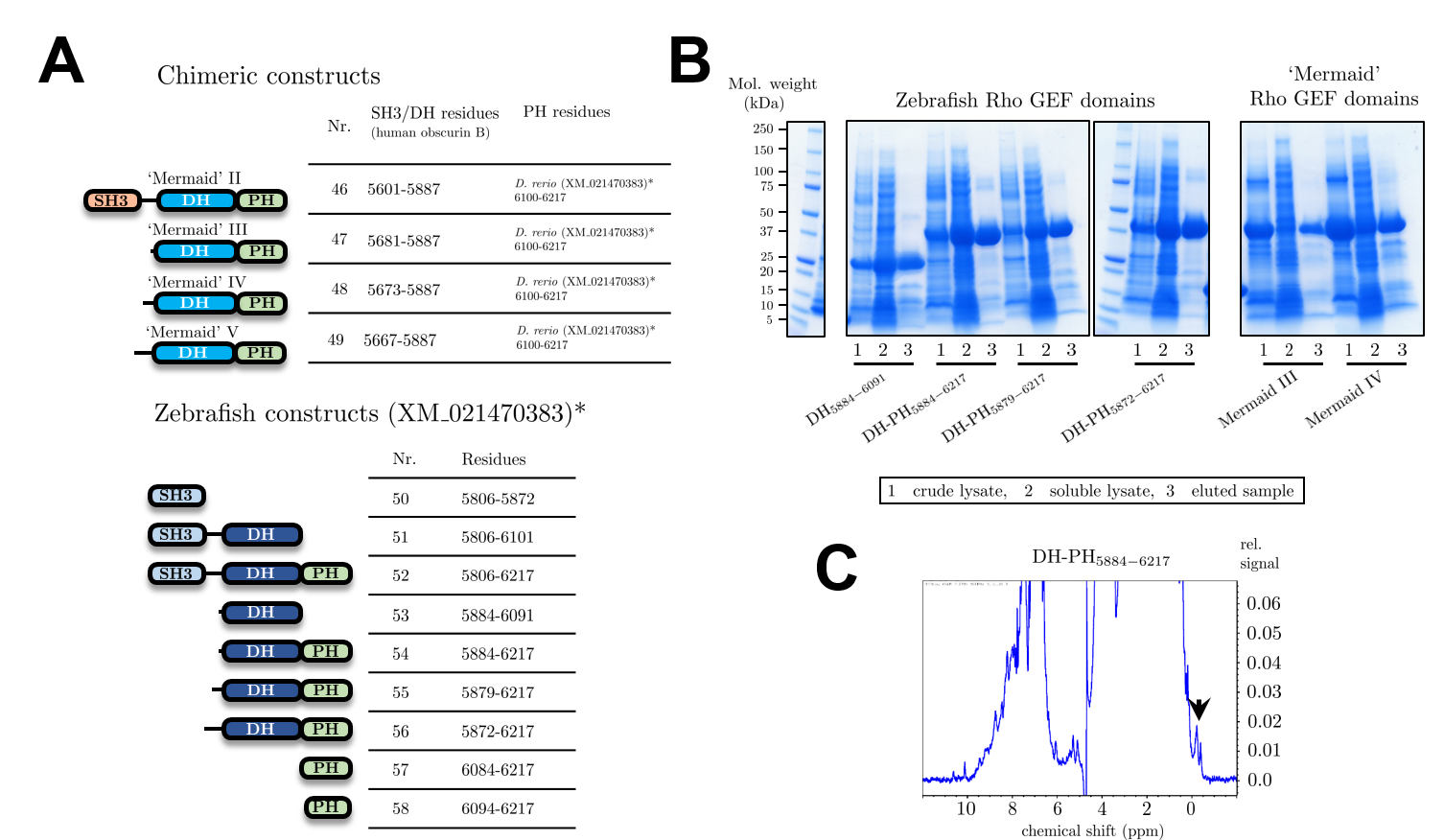

Supplement: S10 Fig — A, construct design. The unique Uniprot-identifier for each protein is given in brackets. When the protein was not available on Uniprot (*), the NCBI-Reference sequence is given instead. B, SDS-PAGE analysis after affinity purification of selected zebrafish and chimeric obscurin RhoGEF fragments. C, 1D-NMR analysis of purified zebrafish DH-PH5884-6217 fragment. NMR spectrum exhibits wide peak dispersal and peaks below 0 ppm (black arrow), indicating that the protein is folded. (PNG) [file pone.0284453.s010.png]

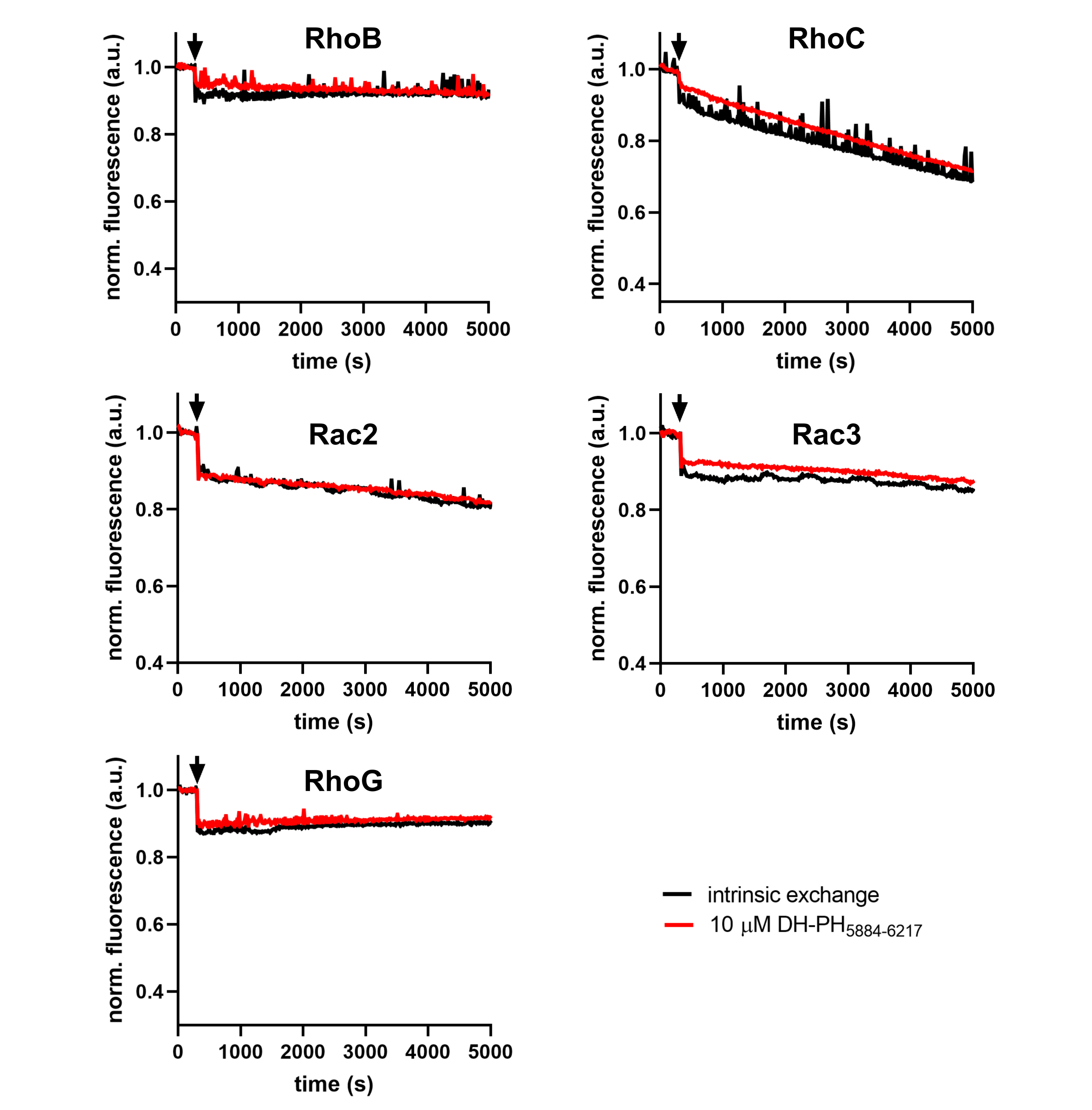

Supplement: S11 Fig — Black arrows indicate addition of buffer/GEF/EDTA. Data represent mean of n = 2–3 experiments. (PNG) [file pone.0284453.s011.png]

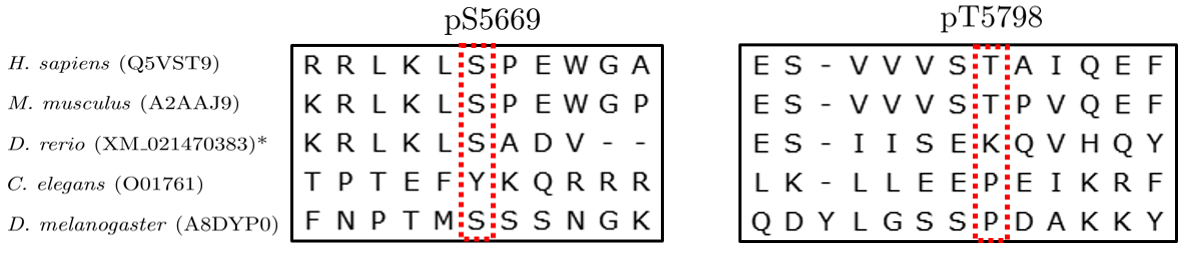

Supplement: S12 Fig — Numbering refers to human obscurin B sequence. (PNG) [file pone.0284453.s012.png]

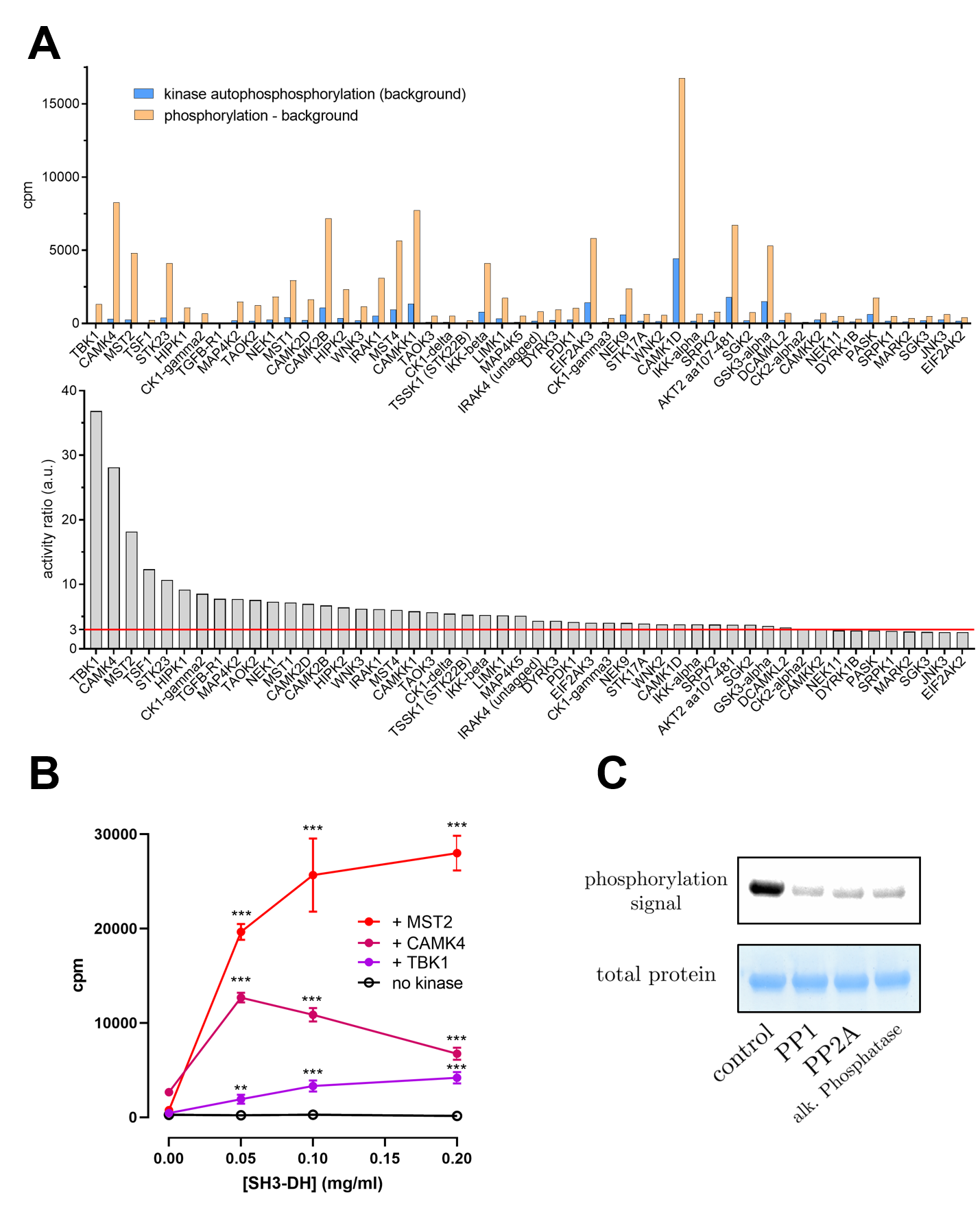

Supplement: S13 Fig — A, (top panel) corrected absolute phosphorylation (orange) of the SH3-DH domain after exposure to a kinase next to the autophosphorylation background signal of that kinase (blue). (Bottom panel) top 50 hits of kinases sorted by the activity ratio of each experiment which considers the corrected absolute phosphorylation of the substrate relative to the phosphorylation background. A value >3 is considered a significant hit. B, validation experiment with same method of top 3 hits in screening assay at different substrate concentrations and n = 3 replicates per concentration (** p < 0.01, *** p < 0.001, students t-test vs no kinase condition). While MST2 addition resulted in strong and saturable phosphorylation, TBK1 led to much lower phosphorylation levels and CaMK4 addition led to an intermediate phosphorylation level exhibiting a biphasic behaviour with phosphorylation levels decreasing at higher substrate concentrations. Since MST2 showed the highest phosphorylation levels of obscurin SH3-DH, we focused on MST2 phosphorylation in all further experiments. C, ProQTM diamond stain signal of MST2-phosphorylated obscurin SH3-DH after addition of no phosphatase (control), phosphatases PP1, PP2A or alkaline Phosphatase. (PNG) [file pone.0284453.s013.png]

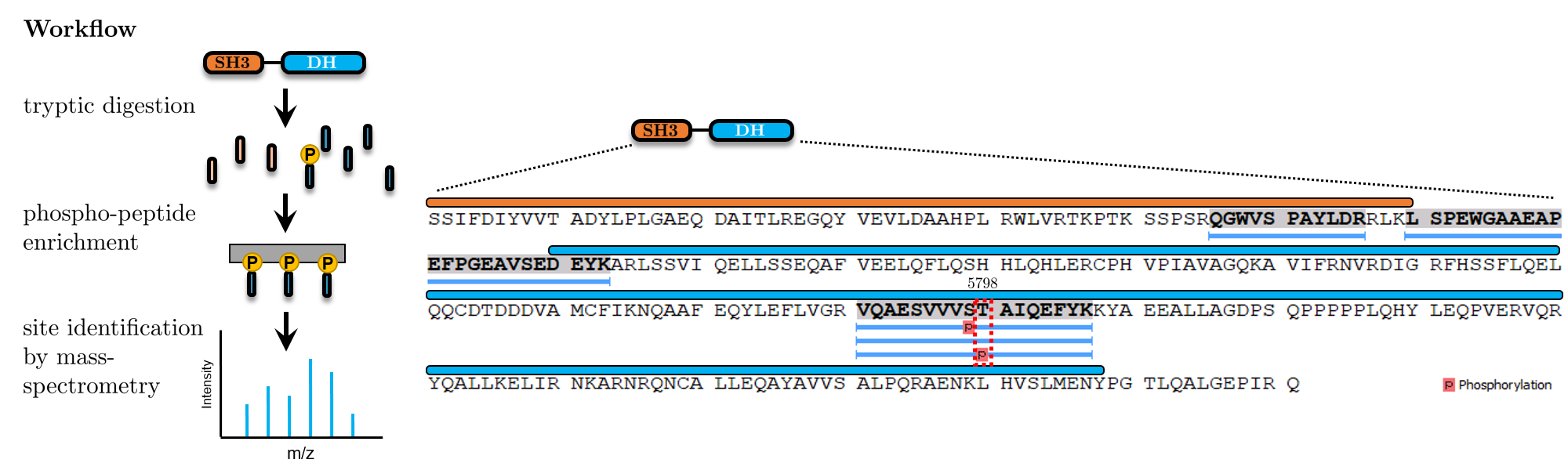

Supplement: S14 Fig — Workflow shown left. Protein sequence and identified peptides and phosphopeptides are shown on the right. Although both pSer5797 and pThr5798 peptides were identified, the precision of the identified site is often associated with an uncertainty of 1 or 2 residues. Since Thr5798 was observed to be phosphorylated in vivo by Potts et al. 2017, we concluded that the phosphorylation site is likely Thr5798. (PNG) [file pone.0284453.s014.png]

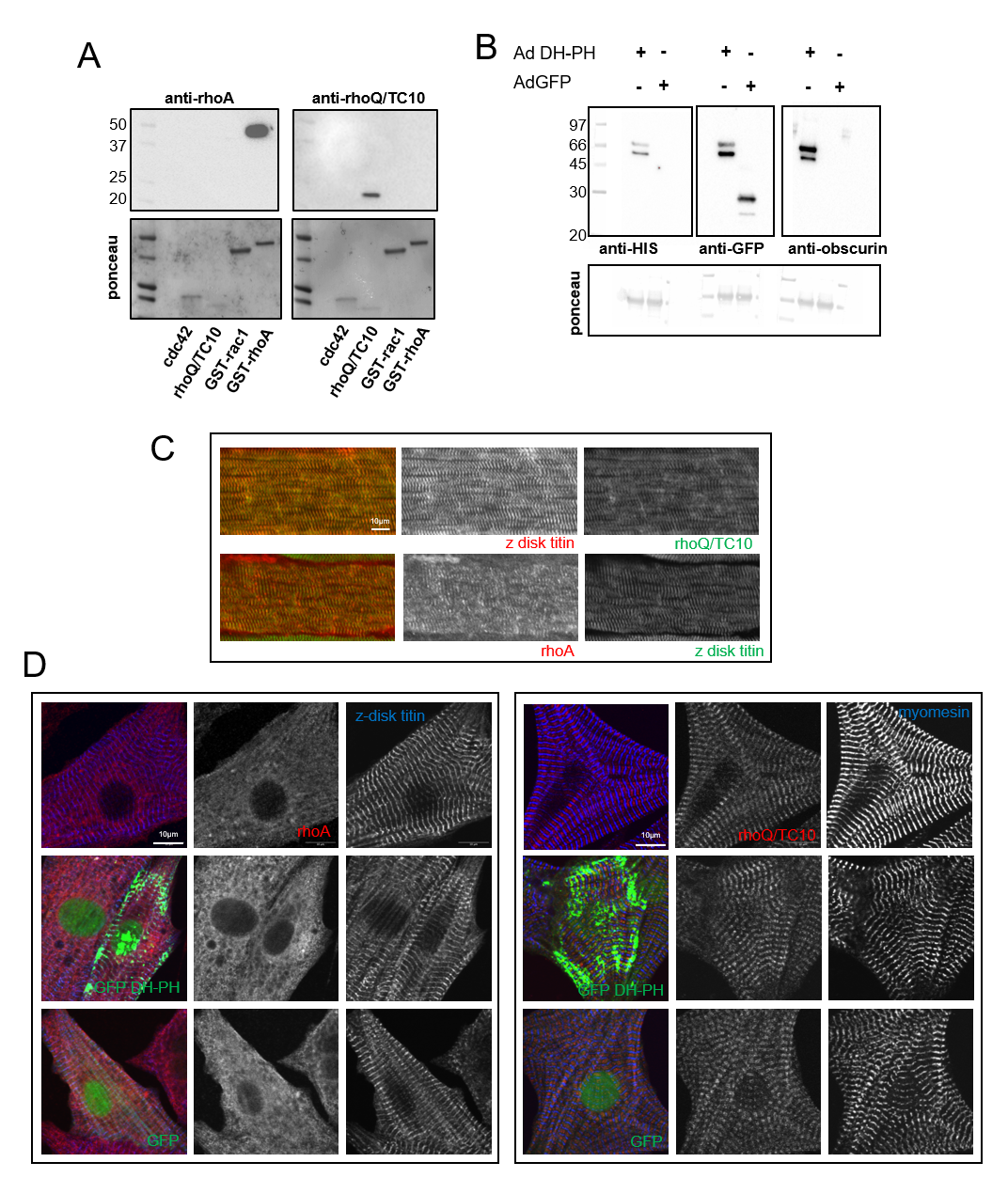

Supplement: S15 Fig — A, Western blot validation of RhoA and RhoQ/TC10 antibodies using recombinant GTPases shows that antibodies specifically bind their target epitope. B, Upper: Western blot confirming expression of obscurin DH-PH. HEK293 cells transduced with adenovirus containing GFP tagged obscurin DH-PH (Ad DHPH) or GFP alone (Ad GFP) and probed with anti-HIS, anti-GFP or anti-obscurin antibodies. Lower: ponceau stain. C, Mouse skeletal muscle (tibialis anterior) stained with upper: rhoQ/TC10 (green) and z-disk titin (red) or lower: rhoA (red) and z-disk titin (green). D, rhoA and rhoQ/TC10 localisation does not change upon overexpression of GFP tagged obscurin DH-PH in neonatal rat cardiomyocytes. Upper row: untransduced cells. Middle row (GFP DH-PH): cardiomyocytes transduced with adenovirus containing GFP obscurin DH-PH. Bottom row (GFP): cardiomyocytes transduced with adenovirus containing GFP only. L: rhoA (red), z-disk titin (blue). R: rhoQ/TC10 (red), myomesin (blue). (PNG) [file pone.0284453.s015.png]

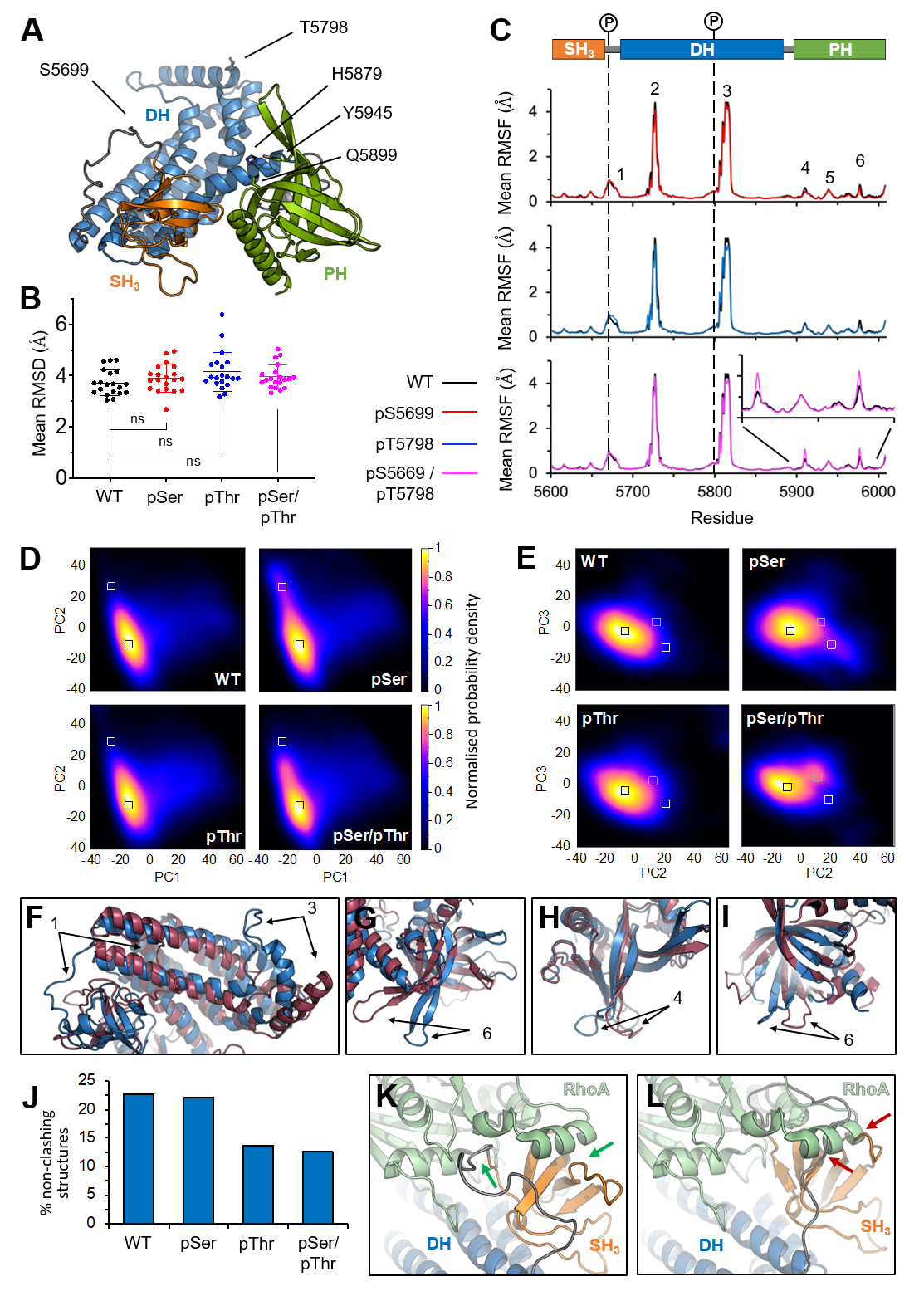

Supplement: S16 Fig — A, Structure of Obscurin SH3DHPH domain triplet predicted using AlphaFold2. The SH3, DH and PH domains are shown in orange, blue and green, respectively. The phosphorylated residues and conserved residues required for RhoGEF activity are labelled and shown as sticks. B, plot showing mean root-mean-squared deviation (RMSD) of structures across the simulations for WT obscurin SH3DHPH and phosphorylated at either serine 5669 (pSer), threonine 5798 (pThr), or both (pSer/pThr). C, plot of the mean root mean squared fluctuation (RMSF) per residue for the three modified proteins compared to WT. D, principal component (PC) 1 and 2 heatmaps for the four molecular species. E, PC 2 and 3 heatmaps for the four molecular species. F-I, structures representing the minima (blue) and maxima (red) for PC2 (F-G) and PC3 (H-I). Arrows indicate the location of the numbered RMSF peaks in C. J, The percentage of structures in the molecular dynamic trajectories for each molecular species that do not clash with RhoA when aligned with Dbs in the Dbs-RhoA crystal structure. Representative models are shown of obscurin SH3DHPH in a binding-compatible (K) and binding-incompatible (L) conformation with RhoA (light green). Arrows indicate clashing (red) and non-clashing (green) regions. (PNG) [file pone.0284453.s016.png]

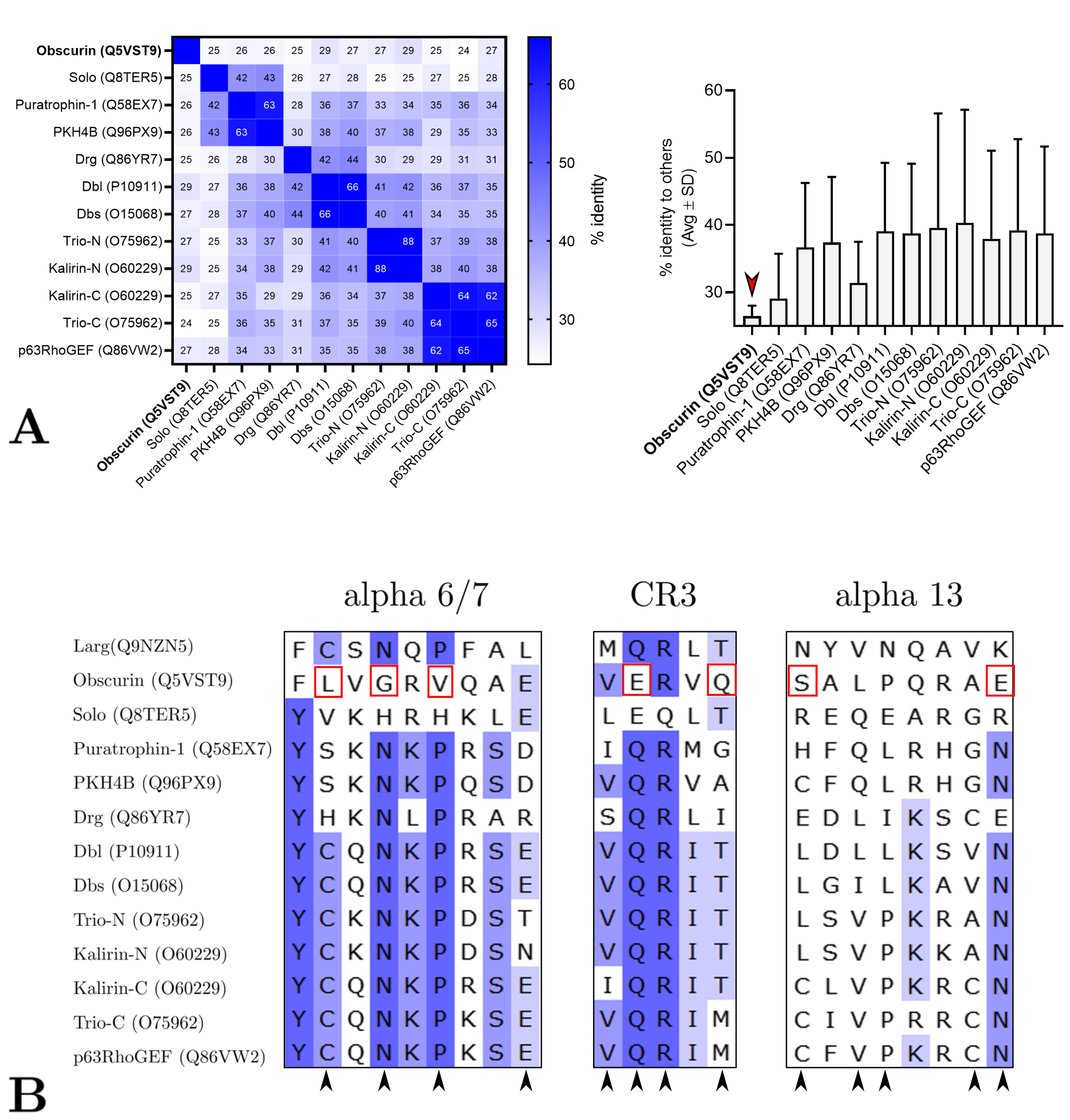

Supplement: S17 Fig — A, multiple sequence alignments of the DH-PH domain sequences of all trio-subfamily members. Heatmap of the percent identity values on the left shows that obscurin is the only GEF that does not cluster (orange squares) with other subfamily members. We defined clusters as largest possible square neighborhoods along the diagonal that have at least 35% sequence identity. This is confirmed by plotting the average identity values for each GEF depicted in the right bar graph, showing that obscurin has the least average identity to other members of the trio-subfamily. B, analysis of key residues in the DH domain that have been identified to be constitutively involved in the interaction with GTPases from all Rho-subfamily members (Rho, Rac, Cdc42) in 13 DH/GTPase complex structures (Jaiswal et al. 2013). From 30 such functionally important and highly conserved residues (black arrowheads), obscurin shows significant differences at 7 of those residues (highlighted by red squares) in the regions alpha 6/7, CR3 and alpha 13 of the DH domain. (PNG) [file pone.0284453.s017.png]

**Fig 1C**

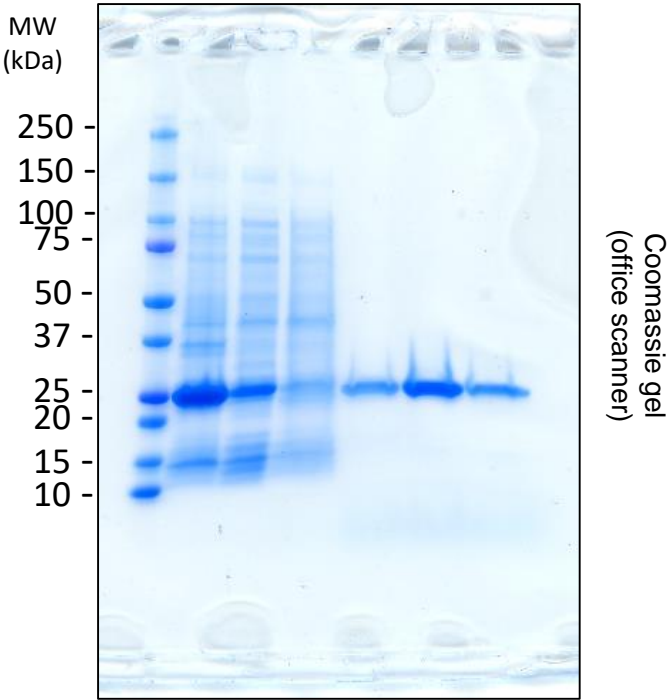

**Fig 4A**

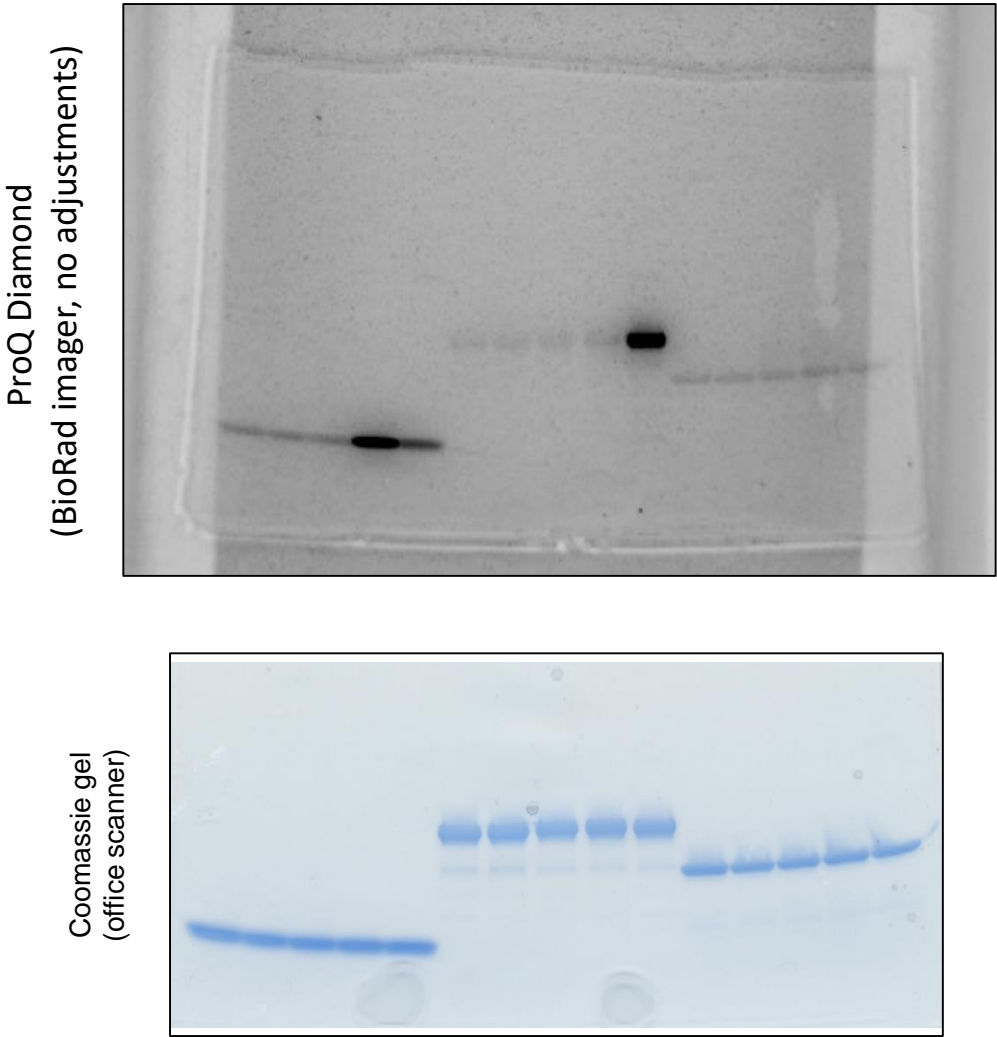

**Fig 4B**

**S13 Fig panel C**

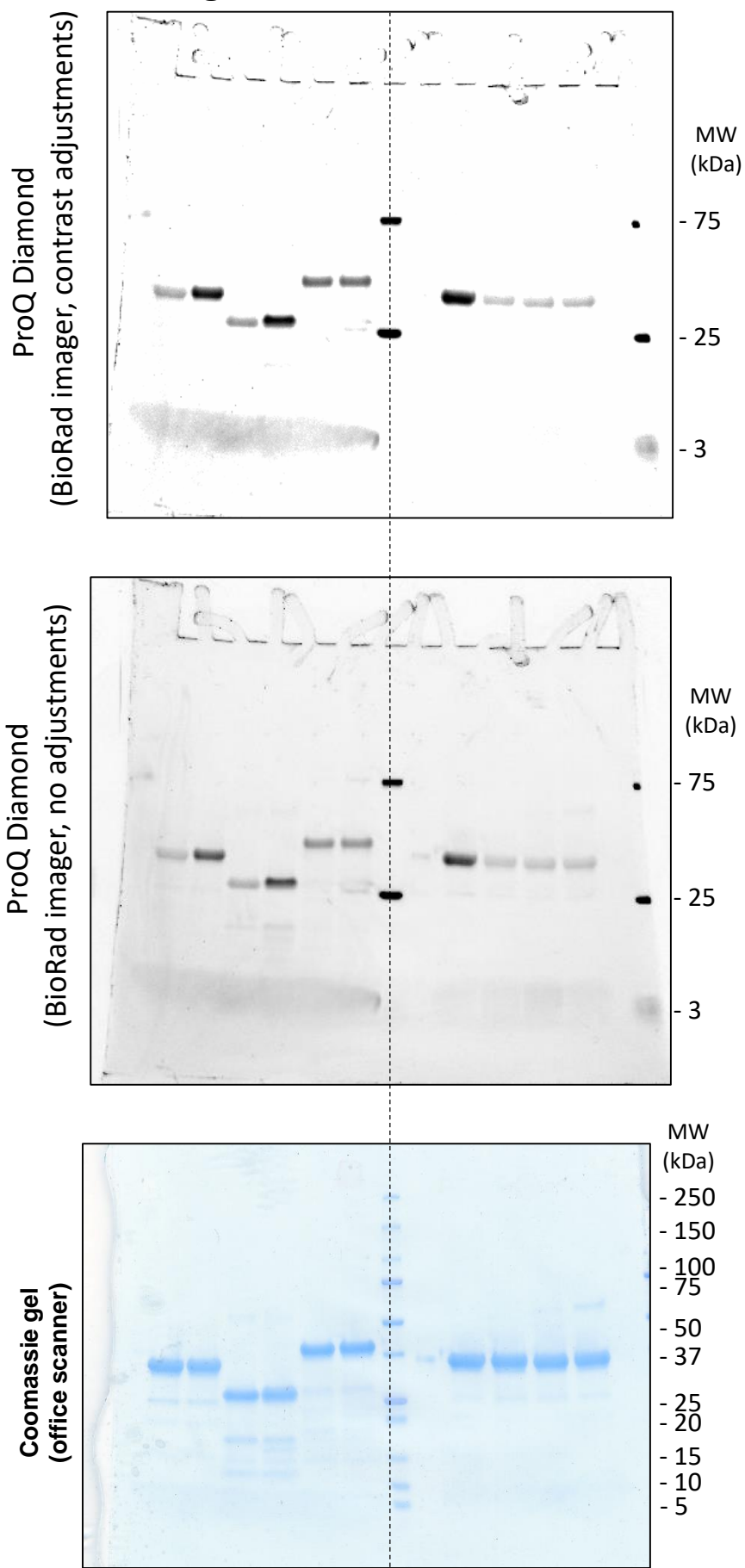

S1 Fig

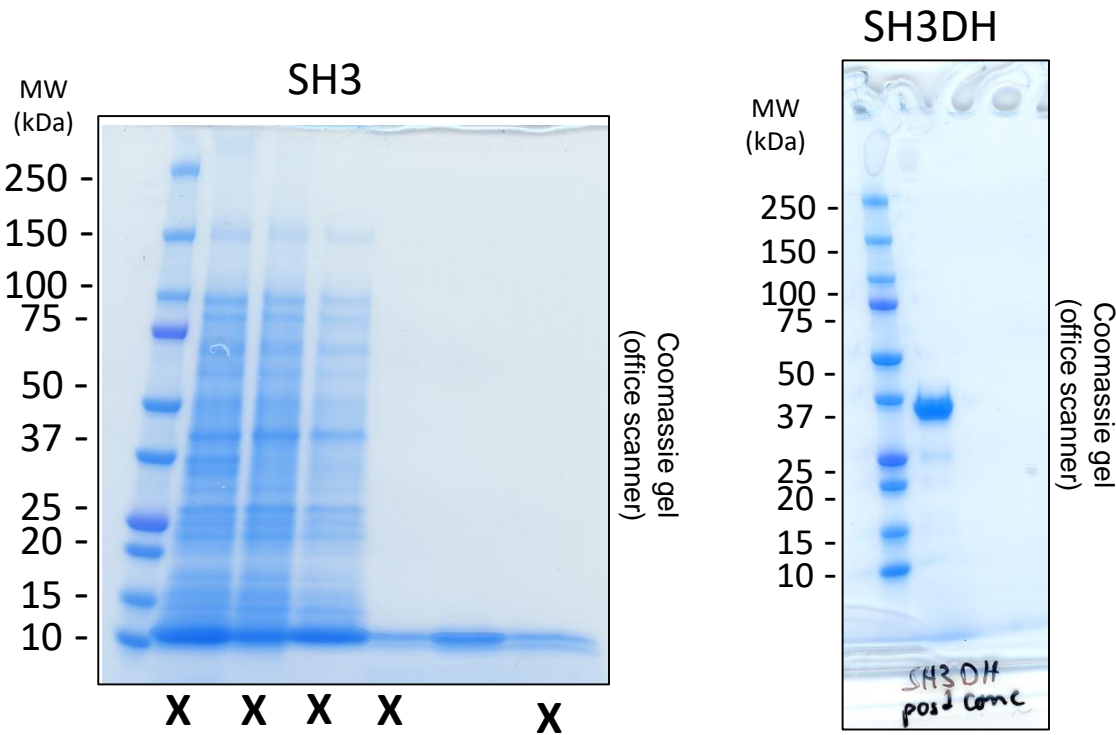

S4 Fig

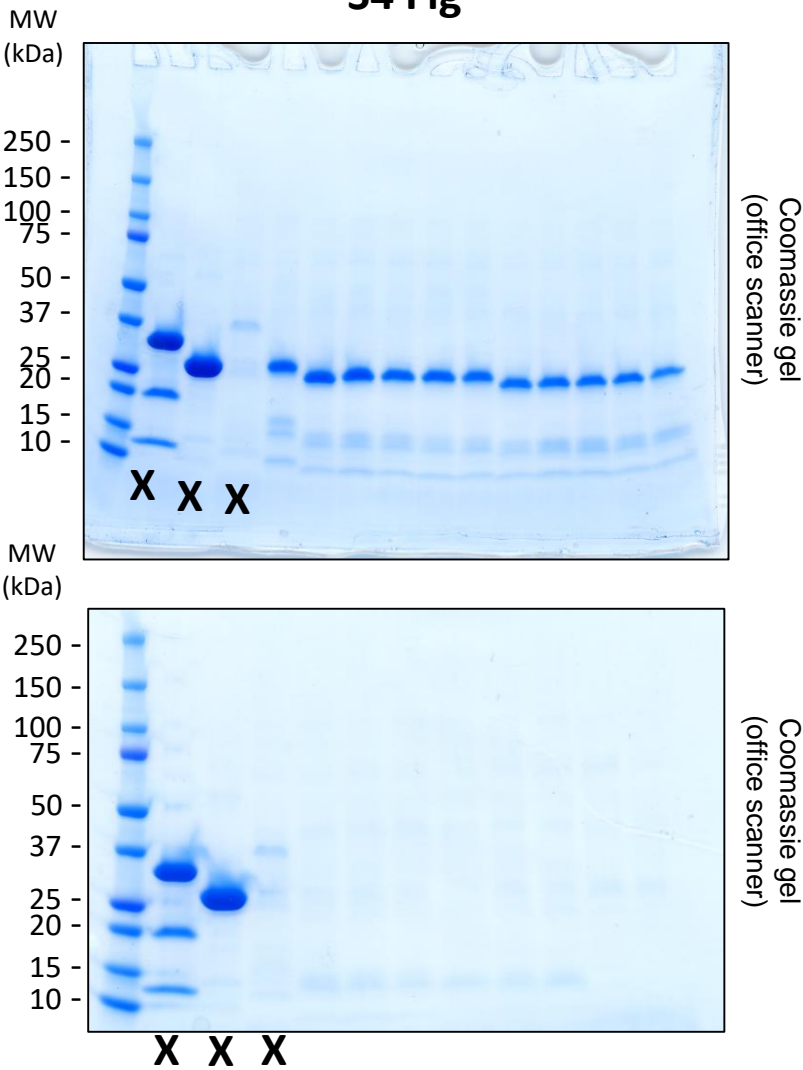

**S5 Fig**

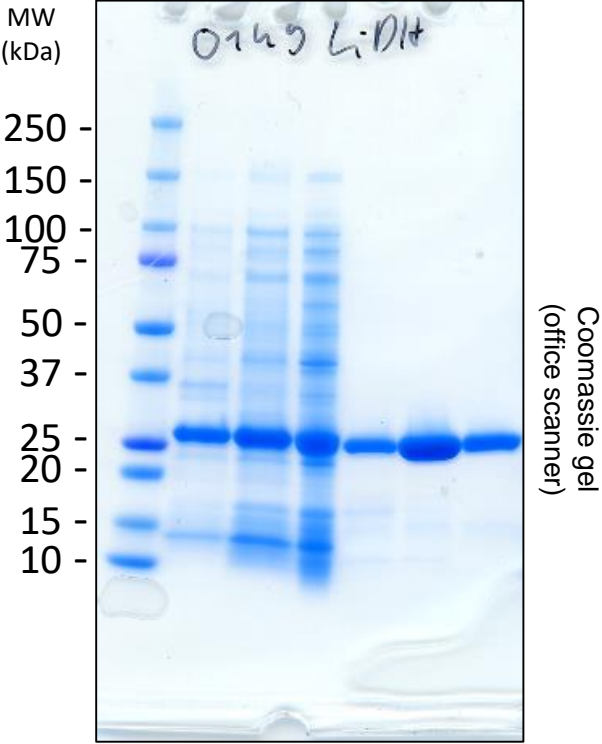

**S7 Fig**

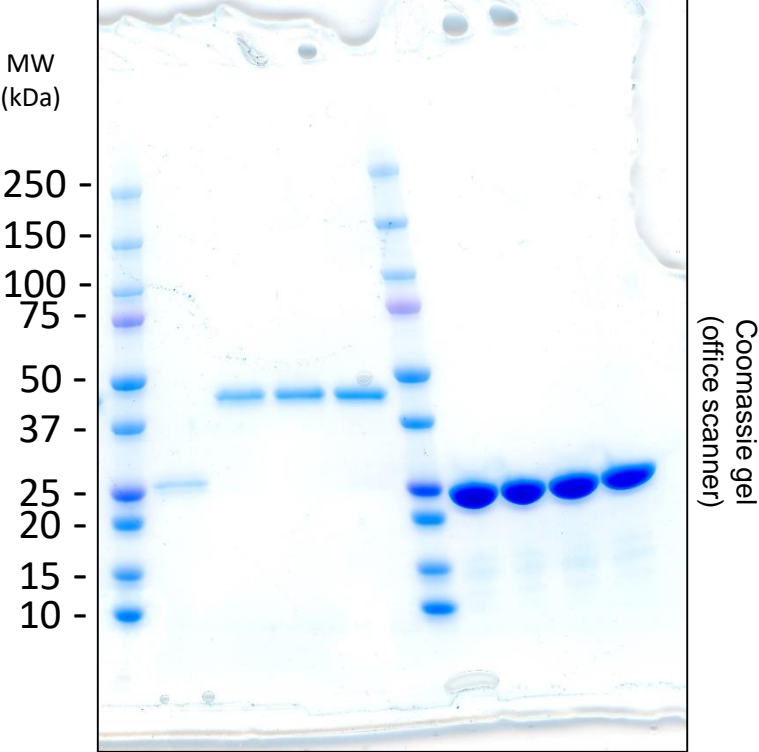

**S10 Fig, panel B**

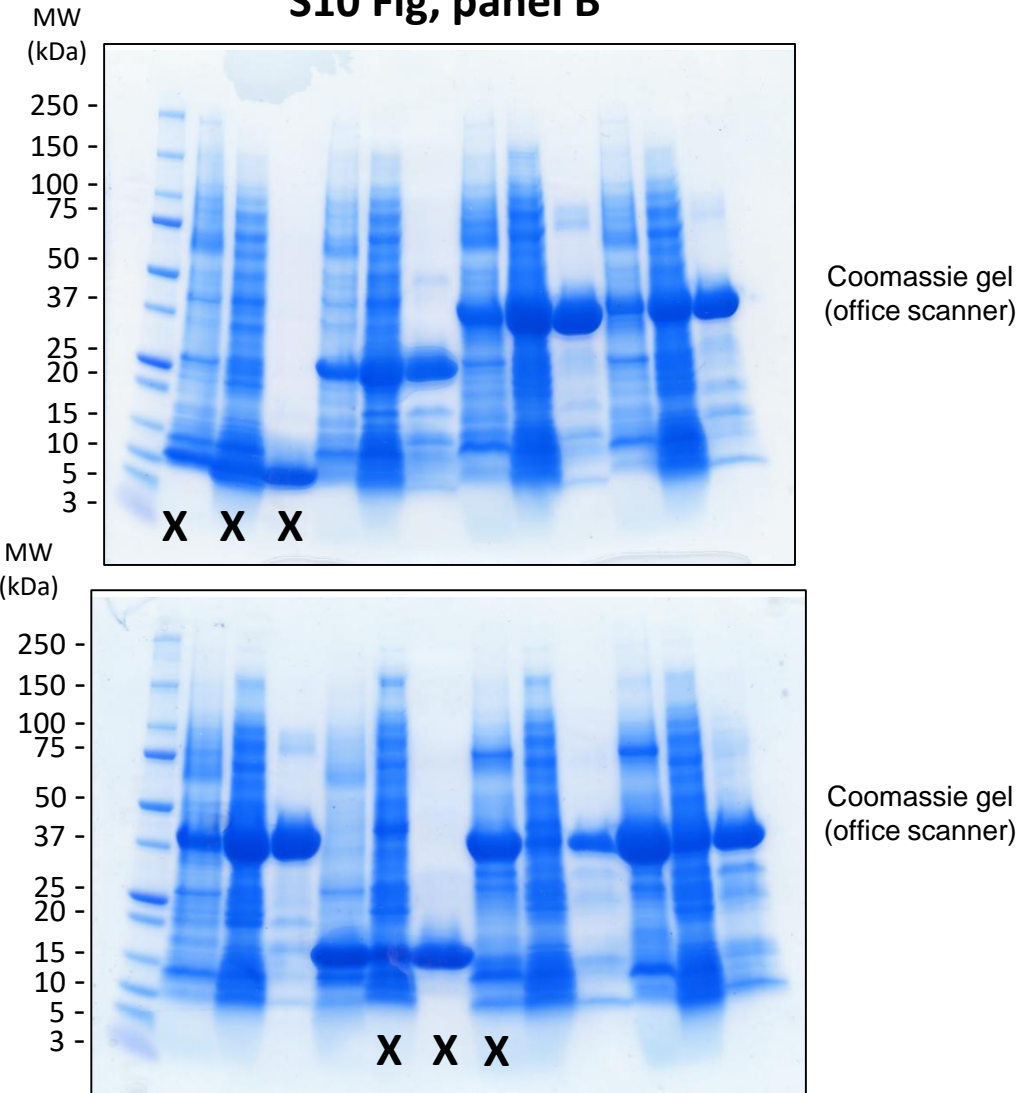

**S14 Fig, panel A**

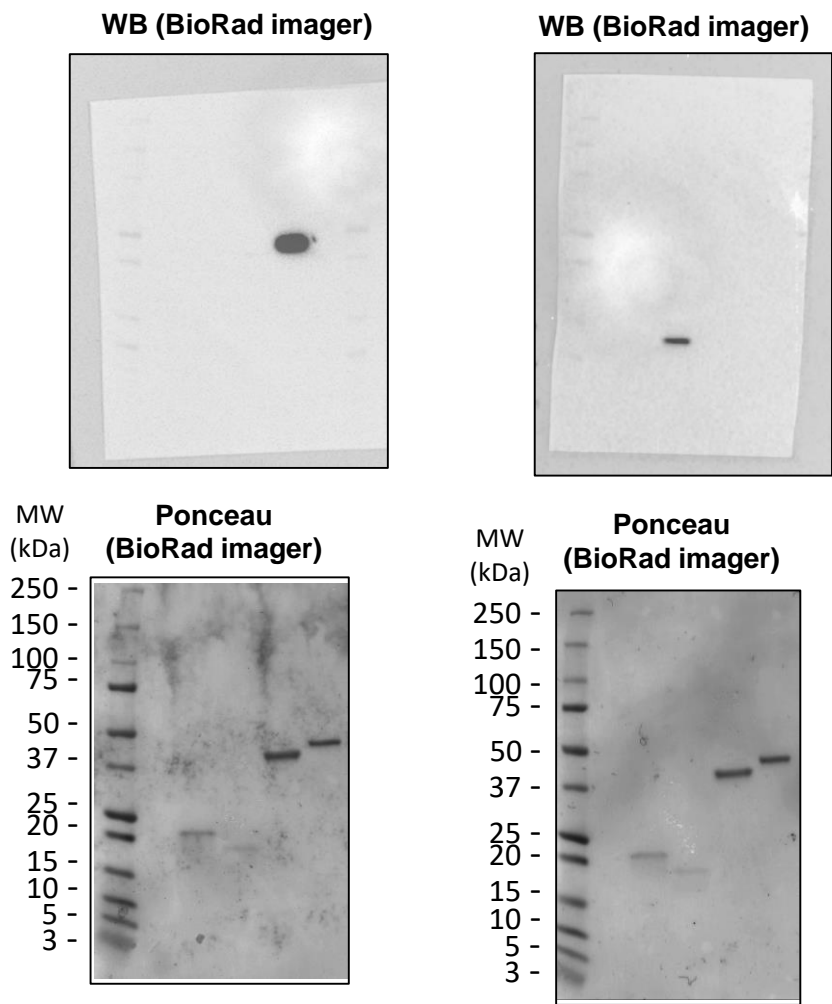

**S14 Fig, panel B**

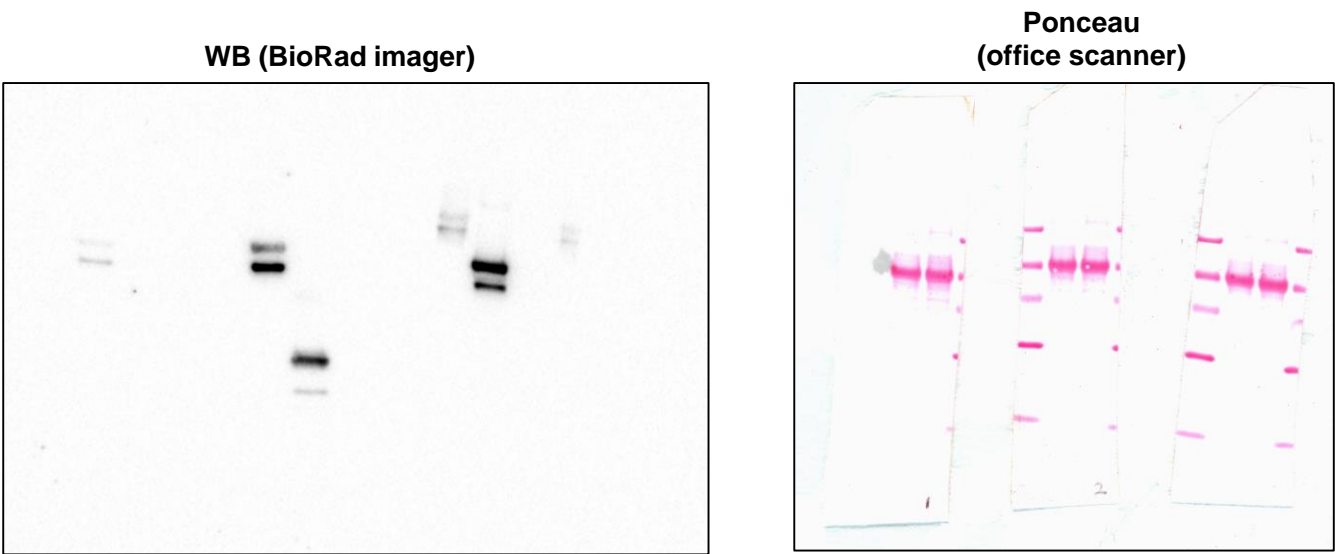

Supplement: S1 Raw images — (PDF) [file pone.0284453.s018.pdf]
