# Supplementary material for: Obscurin Rho GEF domains are phosphorylated by MST-family kinases but do not exhibit nucleotide exchange factor activity towards Rho GTPases in vitro
Source: PLoS One. 2023 Apr 20;18(4):e0284453. doi: 10.1371/journal.pone.0284453 (PMC10118190; doi:10.1371/journal.pone.0284453)
Supplement: S1 Table — (DOCX) [file pone.0284453.s019.docx]

**S1 Table**: **Primers used in this study.**

| **Primer** | **Sequence** | **Tm (°C)** | **Comments** |
| --- | --- | --- | --- |
| 4 | CACGCGTGGATCCTTActggcggatgggctc | 66 | Obscurin 5899 reverse,  5’ overlap with pET6HtevC2 |
| 7 | TATTTTCAGGGCTCGAGCaagctgtcacctgagtgg | 64 | Obscurin 5667 forward,  5’ overlap with pET6HtevC2 |
| 8 | TATTTTCAGGGCTCGAGCcctggggaggctg | 62 | Obscurin 5681 forward,  5’ overlap with pET6HtevC2 |
| 9 | TATTTTCAGGGCTCGAGCtctgaagacgaatac  aaggc | 62 | Obscurin 5686 forward,  5’ overlap with pET6HtevC2 |
| 10 | TATTTTCAGGGCTCGAGCgaatacaaggcaag  gctgag | 63 | Obscurin 5689 forward,  5’ overlap with pET6HtevC2 |
| 11 | TATTTTCAGGGCTCGAGCaaggcaaggctgagctc | 65 | Obscurin 5691 forward,  5’ overlap with pET6HtevC2 |
| 12 | CACGCGTGGATCCTTActccatgagggacacgtg | 65 | Obscurin 5884 reverse,  5’ overlap with pET6HtevC2 |
| 13 | CACGCGTGGATCCTTAggtgcctgggtagttctc | 65 | Obscurin 5889 reverse,  5’ overlap with pET6HtevC2 |
| 14 | CACGCGTGGATCCTTActgcagggtgcctg | 63 | Obscurin 5891 reverse,  5’ overlap with pET6HtevC2 |
| 15 | CACGCGTGGATCCTTActcgcccagggc | 62 | Obscurin 5895 reverse,  5’ overlap with pET6HtevC2 |
| 16 | TATTTTCAGGGCTCGAGCgagcccatccg | 63 | Obscurin 5895 forward,  5’ overlap with pET6HtevC2 |
| 17 | TATTTTCAGGGCTCGAGCcacttcatcgtgtgg | 63 | Obscurin 5901 forward,  5’ overlap with pET6HtevC2 |
| 18 | CACGCGTGGATCCTTAacgctgctggatgc | 62 | Obscurin 6005 reverse,  5’ overlap with pET6HtevC2 |
| 19 | CACGCGTGGATCCTTAaggcagggccagac | 64 | Obscurin 6009 reverse,  5’ overlap with pET6HtevC2 |
| 20 | CACGCGTGGATCCTTAccgccacacaggc | 64 | Obscurin 6012 reverse,  5’ overlap with pET6HtevC2 |
| 21 | tgggtagttctccatgagg | 63 | Obscurin 5887 reverse |
| 22 | ctccatgagggacacg | 62 | Obscurin 5884 reverse |
| 23 | ctcgcccagggc | 62 | Obscurin 5895 reverse |
| 24 | CGTGTCCCTCATGGAGggctatgacgggaatctc | 62 | Dbs 787 forward, 5’ overlap with  human obscurin |
| 25 | CACGCGTGGATCCTTAttctctacaagcctgcagc | 64 | Dbs 921 reverse, 5’ overlap with  pET6HtevC2 |
| 26 | CGTGTCCCTCATGGAGgggtttgatgaaaacattgag | 63 | Trio 1475 forward, 5’ overlap with  human obscurin |
| 27 | CAGGCCCTGGGCGAGctcatcctacaggaatc | 64 | Trio 1486 forward, 5’ overlap with  human obscurin |
| 28 | CACGCGTGGATCCTTAcgtccgctcctggatg | 65 | Trio 1594 reverse, 5’ overlap with  pET6HtevC2 |
| 29 | CTCATGGAGAACTACCCAgg | 64 | Obscurin (chicken) 7963 forward,  5’ overlap with human obscurin |
| 30 | CACGCGTGGATCCTTAgtctggagggatccagac | 64 | Obscurin (chicken) 8096 reverse,  5’ overlap with pET6HtevC2 |
| 31 | CTCATGGAGAACTACCCAgccaatc | 69 | Obscurin (zebrafish) 6090  forward, 5’ overlap with human  obscurin |
| 32 | CACGCGTGGATCCTTAatctggagagcaccatgttg | 64 | Obscurin (zebrafish) 6217  reverse, 5’ overlap with  pET6HtevC2 |

Capitalized bases in sequences indicate overlap regions used for HiFi-DNA-Assembly.
