# Supplementary material for: Obscurin Rho GEF domains are phosphorylated by MST-family kinases but do not exhibit nucleotide exchange factor activity towards Rho GTPases in vitro
Source: PLoS One. 2023 Apr 20;18(4):e0284453. doi: 10.1371/journal.pone.0284453 (PMC10118190; doi:10.1371/journal.pone.0284453)
Supplement: S1 Text — (PDF) [file pone.0284453.s020.pdf]

Info Sheet KinaseFinder
(Version 16)

Purpose of the KinaseFinder Service

The KinaseFinder Service is intended to identify protein kinases, which accept a given test sample (protein or biotinylated peptide) as a substrate in ProQinase´s radiometric ^{33}PanQinase® Assay.

1. Submission of Test Samples

1.1 Protein Samples

Please provide the indicated volumes of a 1 mg/ml stock solution in H_2O or 50 mM HEPES, pH 7.5. Glycerol up to 10%, beta-mercaptoethanol/DTT up to 5 mM and NaCl up to 300 mM are tolerable. The buffer must not contain detergents or imidazole above 50 mM since these will interfere with the assay. If your test sample is not soluble in aqueous solution please contact us. We recommed to ship samples on dry ice to avoid damage of the proteins during transport.

Primary screening will be performed at 5 µg/50 µl sample assay concentration (100 µg/ml).

Hit confirmation will be performed at 2.5 µg/50 µl, 5 µg/50 µl and 10 µg/50 µl sample assay concentration.

Please note: The sample(s) may be returned at customer´s expense. From the date of report, the sample(s) will be stored at -20° Celsius for 6 months and will then be discarded without further notice.

KinaseFinder with 1 assay plate (Tyr version):

- 1 ml ⇨ primary hit finding and hit confirmation with max. 3 hits
- 1.5 ml ⇨ primary hit finding and hit confirmation with max. 7 hits

KinaseFinder with 3 assay plates (Ser/Thr version):

- 2.40 ml ⇨ primary hit finding and hit confirmation with max. 3 hits
- 3.25 ml ⇨ primary hit finding and hit confirmation with max. 7 hits

KinaseFinder with 4 assay plates (complete version):

- 3.25 ml ⇨ primary hit finding and hit confirmation with max. 3 hits
- 3.75 ml ⇨ primary hit finding and hit confirmation with max. 7 hits

<u>Protein stock solutions of other concentrations</u>:

Primary screening will be performed at an sample assay concentration 10 fold lower than the provided sample stock solution concentration.

Hit confirmation will be performed at sample assay concentrations 20-fold, 10-fold and 5-fold lower than the sample stock solution concentration.

1.2 Biotinylated Peptide Samples

Please provide a 200 µM stock solution of the biotinylated peptide, depending on the assay setup, see section 2) in H_2O or 50 mM HEPES, pH 7.5. If your test sample is not soluble in aqueous solution please contact us. Volumes required are a tenth of the volumes indicated above for protein test samples. We recommed to ship samples on dry ice to avoid damage of the peptides during transport.

Primary screening will be performed at 1 µM sample assay concentration.

Hit confirmation will be performed at 1 µM, 0.5 µM and 0.25 µM sample assay concentration.

Please note: The sample(s) may be returned at customer´s expense. From the date of report, the sample(s) will be stored at -20° Celsius for 6 months and will then be discarded without further notice.

<u>Peptide stock solutions of other concentrations:</u>

Primary screening will be performed at a sample assay concentration which is 200-fold lower than the sample stock solution concentration.

Hit confirmation will be performed at a sample assay concentration which is 200-fold, 400-fold and 800-fold lower than the sample stock solution concentration.

Please note: Testing of biotinylated peptides above 1 µM assay concentration is not possible, due to the limited binding capacity of the streptavidine-coated HTS Plus FlashPlates.

2. Assay Conditions

The KinaseFinder experiments are performed with a radiometric filter plate assay (protein samples) or a radiometric assay based on streptavidin-coated FlashPlate® HTS PLUS plates (biotinylated peptide samples).

2.1 Phosphorylation reaction

The samples are tested against each kinase in singlicate in a 96-well polypropylene microtiter plate in a 50 µl reaction volume. The reaction cocktail is pipetted in the following order:

- 10 µl of kinase
- 40 µl of buffer/ATP/test sample mixture

The reaction cocktail contains 60 mM HEPES-NaOH, pH 7.5, 3 mM $MgCl_2$, 3 mM $MnCl_2$, 3 µM Na-orthovanadate, 1.2 mM DTT, 1 µM ATP/[γ-^{33}P]-ATP (approx. 7.5 x 10^{05} cpm per well), protein kinase (1-300 ng/50µl, according to the ^{33}PanQinase® screening assay concentration of the respective kinase/kinase lot) and the protein/peptide of interest.

All PKC assays (except the PKC-mu and the PKC-nu assay) additionally contained 1 mM $CaCl_2$, 4 mM EDTA, 5 µg/ml Phosphatidylserine and 1 µg/ml 1,2-Dioleyl-glycerol.

The CAMK1D, CAMK2A, CAMK2B, CAMK2D, CAMK4, CAMKK1, CAMKK2, DAPK2, EEF2K, MYLK, MYLK2 and MYLK3 assays additionally contained 1 µg/ml Calmodulin and 0.5 mM $CaCl_2$.

The PRKG1 and PRKG2 assays additionally contained 1 µM cGMP.

The DNA-PK assay additionally contained 2.5 µg/ml DNA.

The reaction cocktails are incubated at 30° C for 60 minutes.

Subsequently, depending on the type of test sample (protein or biotinylated peptide), the reaction cocktails are processed differently (see below).

2.2 Detection of phosphorylated proteins

Reaction cocktails with a protein or peptide as potential substrate are stopped with 20 µl of 10% (v/s) H_3PO_4. Subsequently, the reaction cocktails are transferred to pre-wetted 96-well filter plates (Millipore MSFC glass filter), followed by 10 min incubation at room temperature. Subsequently, the plates are washed three times with 250 µl of 150 mM H_3PO4 and once with 20 µl of 100% ethanol. After drying the plates for 30 min at 40° C, 50 µl of scintillator are added and incorporation of $^{33}P_i$ (“counting of cpm”) is determined with a microplate scintillation counter.

2.3 Detection of phosphorylated, biotinylated peptides

Reaction cocktails with a biotinylated peptide are stopped with 20 µl of 4.7 M NaCl/35 mM EDTA. Subsequently, the reaction cocktails are transferred to streptavidin-coated FlashPlate® HTS PLUS plates followed by 30 min incubation at room temperature on a shaker. Then, the plates are washed three times with 250 µl of 0.9% NaCl. Incorporation of $^{33}P_i$ (“counting of cpm”) is determined with a microplate scintillation counter.

3. Evaluation of Raw Data

For evaluation of the results of the filter assay (testing of protein or peptide test samples), kinase autophosphorylation has previously been determined in three independent experiments and a mean autophosphorylation value has been calculated for each kinase. These values (normalized on the radioactivity input of the current experiment) are subtracted from the corresponding raw data obtained with each kinase. Moreover, the median of three background values (sample protein without enzyme) is subtracted from the raw data.

For evaluation of the results from the streptavidin-coated FlashPlate® HTS PLUS plate assay, the activity value (raw counts of the kinase assay plus biotinylated sample peptide as measured in the FlashPlate® HTS PLUS plate assay), the corresponding kinase background value (kinase w/o sample peptide) and the median of three background values of the sample peptide (sample petide w/o enzyme) are measured. The median of three background values is subtracted from the raw data of the results of the testings of biotinylated sample peptide plus kinase.

4. Hit confirmation

Hits detected in a KinaseFinder experiment can be verified in a hit confirmation experiment, where the test sample will be tested at three different concentrations in triplicate (see section 1.1 and 1.2) at enzyme concentrations according to the standard ^{33}PanQinase® screening assay concentration of the respective kinase/kinase lot.

5. Protein Kinases

All kinases currently available for the KinaseFinder Service are listed on the following pages.

5.1 Ser/Thr Kinases

No.	Kinase Name
1	ACV-R1
2	ACV-R1B
3	ACV-R2A
4	ACV-R2B
5	ACV-RL1
6	AKT1
7	AKT2
8	AKT3
9	AMPK-alpha1
10	ARK5
11	ASK1
12	Aurora-A
13	Aurora-B
14	Aurora-C
15	BMPR1A
16	B-RAF VE
17	B-RAF wt
18	BRSK1
19	BRSK2
20	BUB1B
21	CAMK1D
22	CAMK2A
23	CAMK2B
24	CAMK2D
25	CAMK2G
26	CAMK4
27	CAMKK1
28	CAMKK2
29	CDC42BPA
30	CDC42BPB
31	CDC7/ASK
32	CDK1/CycA
33	CDK1/CycE
34	CDK16/CycK
35	CDK1CycB1
36	CDK2/CycA
37	CDK2/CycE
38	CDK3/CycE
39	CDK4/CycD1
40	CDK4/CycD3
41	CDK5/p25NCK
42	CDK5/p35NCK
43	CDK6/CycD1
44	CDK6/CycD3
45	CDK7CycH
46	CDK8/CycC
47	CDK9/CycK
48	CDK9/CycT
49	CHK1
50	CHK2
51	CK1-alpha1
52	CK1-delta
53	CK1-epsilon
54	CK1-gamma1
55	CK1-gamma2
56	CK1-gamma3
57	CK2-alpha1
58	CK2-alpha2
59	CLK1
60	CLK2
61	CLK3
62	CLK4

No.	Kinase Name
63	COT
64	DAPK1
65	DAPK2
66	DAPK3
67	DCAMKL2
68	DMPK
69	DNA-PK
70	DYRK1A
71	DYRK1B
72	DYRK2
73	DYRK3
74	DYRK4
75	EEF2K
76	EIF2AK2
77	EIF2AK3
78	ERK1
79	ERK2
80	ERK7
81	GRK2
82	GRK3
83	GRK4
84	GRK5
85	GRK6
86	GRK7
87	GSG2
88	GSK3-alpha
89	GSK3-beta
90	HIPK1
91	HIPK2
92	HIPK3
93	HIPK4
94	HRI
95	IKK-alpha
96	IKK-beta
97	IKK-epsilon
98	IRAK1
99	IRAK4
100	JNK1
101	JNK2
102	JNK3
103	LIMK1
104	LIMK2
105	LRRK G2019S
106	LRRK2 I2020T
107	LRRK2 R1441C
108	LRRK2 wt
109	MAP3K1
110	MAP3K10
111	MAP3K11
112	MAP3K7/MAP3K7IP1
113	MAP3K9
114	MAP4K2
115	MAP4K4
116	MAP4K5
117	MAPKAPK2
118	MAPKAPK3
119	MAPKAPK5
120	MARK1
121	MARK2
122	MARK3
123	MARK4
124	MEK1 wt

No.	Kinase Name
125	MEK2
126	MEK5
127	MEKK2
128	MEKK3
129	MELK
130	MINK1
131	MKK4
132	MKK6SDTD
133	MKK7
134	MKNK1
135	MKNK2
136	MLK4
137	MST1
138	MST2
139	MST3
140	MST4
141	mTOR
142	MYLK
143	MYLK2
144	MYLK3
145	NEK1
146	NEK11
147	NEK2
148	NEK3
149	NEK4
150	NEK6
151	NEK7
152	NEK9
153	NIK
154	NLK
155	p38-alpha
156	p38-beta
157	p38-delta
158	p38-gamma
159	PAK1
160	PAK2
161	PAK3
162	PAK4
163	PAK6
164	PAK7
165	PASK
166	PBK
167	PDK1
168	PHKG1
169	PHKG2
170	PIM1
171	PIM2
172	PIM3
173	PKA
174	PKC-alpha
175	PKC-beta1
176	PKC-beta2
177	PKC-delta
178	PKC-epsilon
179	PKC-eta
180	PKC-gamma
181	PKC-iota
182	PKC-mu
183	PKC-nu
184	PKC-theta
185	PKC-zeta
186	PKC-zeta wt aa184-592

No.	Kinase Name
187	PKMYT1
188	PKN3
189	PLK1
190	PLK3
191	PRK1
192	PRK2
193	PRKD2
194	PRKG1
195	PRKG2
196	PRKX
197	RAF1 YDYD
198	RIPK2
199	RIPK5
200	ROCK1
201	ROCK2
202	RPS6KA1
203	RPS6KA2
204	RPS6KA3
205	RPS6KA4
206	RPS6KA5
207	RPS6KA6
208	S6K
209	S6K-beta
210	SAK
211	SGK1
212	SGK2
213	SGK3
214	SIK1 aa1-350
215	SIK2
216	SLK
217	SNARK
218	SNK
219	SRPK1
220	SRPK2
221	STK17A
222	STK23
223	STK25
224	STK33
225	STK39
226	TAOK2
227	TAOK3
228	TBK1
229	TGFB-R1
230	TGFB-R2
231	TLK1
232	TLK2
233	TSF1
234	TSK2
235	TSSK1
236	TTBK1
237	TTBK2
238	TTK
239	VRK1
240	VRK2
241	WEE1
242	WNK1
243	WNK2
244	WNK3
245	ZAK

5.2 Tyr Kinases

No.	Kinase Name
1	ABL1 T315I
2	ABL1 wt
3	ABL2
4	ACK1
5	ALK F1174L (GST-HIS-tag)
6	ALK L1196M (GST-HIS-tag)
7	ALK wt
8	AXL
9	BLK
10	BMX
11	BRK
12	BTK
13	CSF1-R
14	CSK
15	DDR2 T654M
16	DDR2 wt
17	EGF-R T790M
18	EGF-R wt
19	EPHA1
20	EPHA2
21	EPHA3
22	EPHA4
23	EPHA5
24	EPHA6
25	EPHA7
26	EPHA8
27	EPHB1
28	EPHB2
29	EPHB3
30	EPHB4
31	ERBB2 wt
32	ERBB4
33	FAK
34	FER
35	FES
36	FGF-R1 V561M
37	FGF-R1 wt
38	FGF-R2
39	FGF-R3 K650E
40	FGF-R3 wt
41	FGF-R4
42	FGR
43	FLT3 D835Y
44	FLT3 ITD
45	FLT3 wt
46	FRK
47	FYN
48	HCK
49	IGF1-R
50	INS-R
51	INSR-R
52	ITK
53	JAK1
54	JAK2
55	JAK3
56	KIT D816V
57	KIT T670I
58	KIT wt
59	LCK
60	LTK
61	LYN
62	MATK

No.	Kinase Name
63	MERTK
64	MET T1250M
65	MET wt
66	MET Y1235D
67	MUSK
68	PDGFR-alpha D842V
69	PDGFR-alpha wt
70	PDGFR-beta
71	PYK2
72	RET V804M
73	RET wt
74	RON
75	ROS
76	SRC
77	SRMS
78	SYK
79	TEC
80	TIE2 wt
81	TIE2 Y897S
82	TNK1
83	TRK-A
84	TRK-B
85	TRK-C
86	TXK
87	TYK2
88	TYRO3
89	VEGF-R1
90	VEGF-R2
91	VEGF-R3
92	WEE1
93	YES
94	ZAP70
