# Supplementary material for: Obscurin Rho GEF domains are phosphorylated by MST-family kinases but do not exhibit nucleotide exchange factor activity towards Rho GTPases in vitro
Source: PLoS One. 2023 Apr 20;18(4):e0284453. doi: 10.1371/journal.pone.0284453 (PMC10118190; doi:10.1371/journal.pone.0284453)
Supplement: S2 Text — (DOCX) [file pone.0284453.s021.docx]

**S2 Text**

The following technical specifications were provided by the Metabolomics and Proteomics Laboratory of the Bioscience Technology Facility of the University of York:

*The coomassie stained bands have been analysed for phosphopeptide identification as requested. Briefly, protein was digested with trypsin in-gel, following reduction with DTE and alkylation with iodoacetamide. Peptides were extracted from gel before phosphopeptide enrichment using MagReSyn titanium dioxide microparticles. Enriched phosphopeptides were loaded onto a 50 cm EN C18 PepMap column with elution over a 1 h acquisition driven by a Waters mClass nanoUPLC. Eluted peptides were measured using a Thermo Orbitrap Fusion Tribrid mass spectrometer, with MS spectra acquired in the Orbitrap and MS2 spectra acquired in parallel, following HCD fragmentation, in the linear ion trap.*

*Data were searched against the protein sequence provided, appended with common proteomic contaminants, using PEAKS Studio X Pro. Carbamidomethylation of Cys was set as a fixed modification and oxidation of Met and phosphorylation of Ser, Thr and Tyr were all set as variable.*

*Peptide matches were filtered to require individual peptide assignments to have a -10log10P score of >20, which equates to p<0.01. When assessed at this threshold against a decoy database search, all searches yielded an empirical false discovery rate of <1%.*

*In all three cases the expected protein identification was made. Phosphorylated peptides are also detected in all three samples.*

*Please find a summary of the results attached, listing all identified peptides. There is a separate tab in the file for each sample.*

*When assessing the results please note the final column “Ascore = -10lgP site localisation prob.”. Here you will find the site localisation probability for each PTM detected. (…) The probability for the peptide identification including a single phosphorylation is given in row C as -10lgP ID prob. (…). Looking at column Q you will see (…) the site localisation probability for the phosphorylation to sit at the Ser annotated (…).*

*Below each table of peptide identifications there is a visual representation of the peptides and phosphorylation identified, as also presented below:*


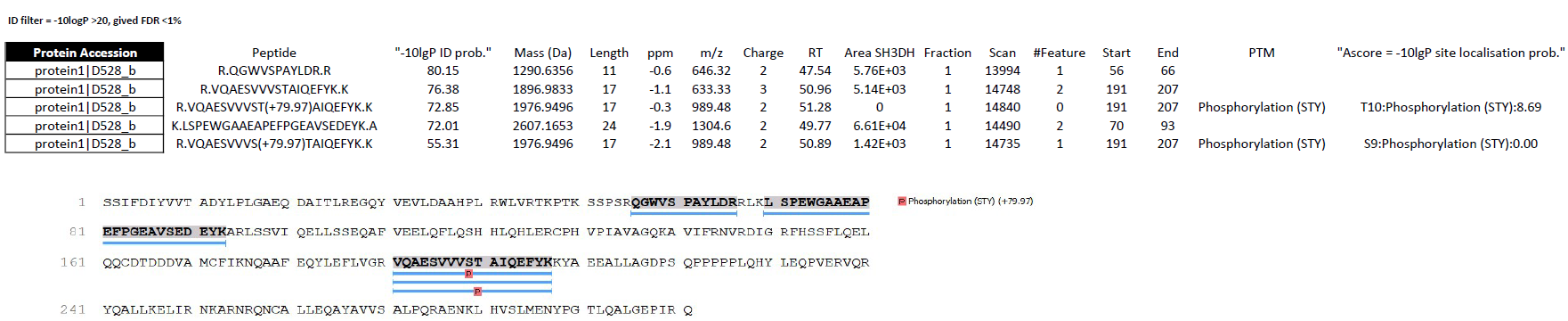


*In these images peptide identifications are also filtered to -10log10P>20. Phosphorylation annotated above the line of amino acids represents those with site localisation Ascores>20.*

*Please note that it is common for site localisation to be more confident were potential phosphosites are more spread out. Where sites are very close together they can be harder to distinguish. Also you should consider the possibility that both sites may be detected within the same spectrum as different phosphorylation on adjacent or near adjacent residues may change the retention time very little and if they are not resolved by LC will have identical MS1 masses and so appear in the same chimeric spectrum.*
