# Supplementary material for: Obscurin Rho GEF domains are phosphorylated by MST-family kinases but do not exhibit nucleotide exchange factor activity towards Rho GTPases in vitro
Source: PLoS One. 2023 Apr 20;18(4):e0284453. doi: 10.1371/journal.pone.0284453 (PMC10118190; doi:10.1371/journal.pone.0284453)
Supplement: S3 Text — (DOCX) [file pone.0284453.s022.docx]

**S3 Text**

To assess whether phosphorylation at Ser5669 and Thr5798 affects the structure and dynamics of the SH3DHPH region of obscurin and could therefore regulate any GEF activity, molecular dynamics simulations were run on WT and phosphorylated, AlphaFold-predicted, models (**S16 Fig, panel A**) of these domains (20 runs per molecular species of 1us each).

There was no difference in the root mean square deviation (RMSD) between the WT and three phosphorylated species tested (phosphorylated at either Ser5669 or Thr5798, or both positions, **S16 Fig, panel B**). A plot of root mean squared fluctuation (RMSF) per residue showed six “peaks” indicating regions of higher flexibility, with peaks 4 and 6 showing a higher fluctuation for the double phosphorylated structure compared to the other three molecular species (**S16 Fig, panel C**).

Principal component analysis of the molecular dynamics trajectories was run to identify differences in the conformation space occupied by each molecular species, with the first 3 principal components (PCs), which described 27% of all the variance, studied in detail. Each molecular species occupied largely the same region of the PCs 1 and 2 heatmap (black box, **S16 Fig, panel D**) but SH3DHPH phosphorylated at Ser5669 also occupied a region at higher PC2 values (grey box). The PC2 maximum structure described in part the hinging of the SH3-DH linker, with an “open” conformation (that would allow binding of RhoGTPases) more frequently populated by SH3DHPH pSer5669 (**S16 Fig, panel F**), along with a difference in the minimum and maximum structures at the hinge region of a two-stranded beta sheet in the PH domain, corresponding to the 6th RMSF peak (**S16 Fig, panel G**).

The four molecular species largely occupy the same regions in the PCs 2 and 3 heatmap (**S16 Fig, panel E**, black box) but SH3DHPH pSer5669 and doubly-phosphorylated SH3DHPH also occupy a region of lower (white box) and higher (grey box) PC3 values, respectively. The main differences in the PC3 minimum and maximum structures are located at residues at the 4th RMSF peak (**S16 Fig, panel H**), near conserved residues involved in RhoGTPase interaction, and the beta turns at residues corresponding to the 6th RMSF peak (**S16 Fig, panel I**).

To quantify if there were any differences in the time spent by each molecular species occupying a conformation favourable to RhoGTPase binding, the structures generated through the MD trajectories were aligned with the crystal structure of the RhoGEF Dbs in complex with RhoA, and structures containing residues that would sterically clash with RhoA identified. While most structures in all trajectories would result in a clash with RhoA, SH3DHPH phosphorylated at Thr5798 singly and in combination with pSer5669 showed a ~36% decrease in conformations that would allow RhoA binding (**S16 Fig, panel J-L**).

These results identify differences in the dynamics and states populated by the different molecular species, but it is currently not possible to assess whether this would affect the ability of obscurin to act as a RhoGEF, or may instead relate to the binding of different ligands.
