# Supplementary material for: Obscurin Rho GEF domains are phosphorylated by MST-family kinases but do not exhibit nucleotide exchange factor activity towards Rho GTPases in vitro
Source: PLoS One. 2023 Apr 20;18(4):e0284453. doi: 10.1371/journal.pone.0284453 (PMC10118190; doi:10.1371/journal.pone.0284453)
Supplement: Attachment — Submitted filename: Response to reviewers.docx [file pone.0284453.s023.docx]

PONE-D-22-28822

Obscurin Rho GEF domains are phosphorylated by MST-family kinases but do not
exhibit nucleotide exchange factor activity towards Rho GTPases in vitro

**General response to both reviewer 1 and reviewer 2:**

*We thank the reviewers for their constructive inputs and suggestions. Since the relevance of testing a full SH3-DH-PH fragment has been pointed out by both reviewers, we have conducted further in vitro experiments to explore the activity of such a fragment. We also tested the influence of P(3,4)IP_2_ as a potentially relevant and physiological co-factor and included some further molecular dynamics simulation data. In agreement with our previous data, we did not detect any activity. Due to limitations in both time and resources available for this project, however, we are not able to conduct further cell biological experiments, although we fully agree with the reviewers that such future work will be necessary to understand the function of the obscurin RhoGEF domains. Despite this limitation, we believe our work represents a carefully conducted biochemical study that will be a useful starting point for others in the field.*

**Reviewer #1: (…)
Specific comments**

**Did the authors try and generate a ‘mermaid’ version containing human SH3, human DH and zebrafish PH domains to obtain soluble protein for subsequent biochemical analyses? Could it be that these domains may require their mutual presence in order to exert biological activity? There is a report that the obscurin PH domain interacts with lipids (e.g. PIP2; Ackermann et al. JMCC 2017). Did the authors try and recapitulate this binding, and/or test if presence of lipids affects the biological RhoGEF activity towards RhoA, Cdc42, Rac1 or RhoQ/TC10?**

*We thank the reviewer for these suggestions and agree that testing the SH3-DH-PH fragment and the influence of phospholipids seems promising. We did indeed clone a chimeric SH3-DH-PH fragment (see mermaid II in Figure S10A). However, in a small scale expression/purification test, this fragment turned out not be soluble. We thus decided to proceed with the zebrafish sequence and successfully purified the zebrafish obscurin SH3-DH-PH fragment. Together with the zebrafish DH-PH fragment we tested the SH3-DH-PH fragment against RhoA and RhoQ both in presence and absence of P(3,4)IP_2_. As described in the revised manuscript and Fig.5, neither of these approaches was successful in achieving GEF activity.*

**Did the authors try and express the full SH3-DH-PH tandem in cells and test for a change to RhoA and RhoQ localization?**

*Based on the data from Ford-Speelman et al. and Coisy-Quivy et al. the DH-PH domains should in principle be sufficient to see an effect. At the time we thus opted for the DH-PH fragment and not the full SH3-DH-PH fragment in cells. Due to the mentioned limitations (see our general response above), we are unable to conduct further cell-biological experiments for this project.*

**Figure 14 only seems to show transduction of neonatal cardiomyocytes? The manuscript states that also adult murine cardiomyocytes were tested?**

*We indeed did the same experiments in AMCs but with the same outcome. We felt these results do not add much to apart from further confirming the inactivity our manuscript so decided against showing them. However, we now explicitly mention that these are not shown.*

**There seem to some experimental results in the supplemental data that are not mentioned in the main text of the manuscript. Specifically, Figure S14 shows several validation experiments (Panels A-B) and immunofluorescence of mouse skeletal muscle (Panel C) that is not mentioned anywhere in the manuscript test.**

*We now explicitly mention the validation experiments in our manuscript and refer to the respective subfigures of Figure S15 (previously S14).*

**It is a bit strange that the authors see RhoA localization in adult skeletal muscles at the Z-disc (Figure S14C). Several manuscripts describe RhoA localization at the M-band in adult tissues, including a manuscript the authors cite (Ford-Speelman et al.). In addition, RhoA localization at M-bands was lost in obscurin knockout muscles (Lange et al. MBoC. 2012), suggesting at least compartmentalisation of RhoA by obscurin. How do the authors explain this discrepancy?**

*We agree with the discrepancy but are unsure as to what exactly the reason for this discrepancy is. One possible explanation could be different specificities of the used antibodies. Both Ford-Speelman et al. and Lange et al. used a RhoA antibody from Santa-Cruz, whereas we used an antibody from Sigma-Aldrich. However, we confirmed antibody specificity with recombinant proteins at least for four RhoGTPases, which neither of the other studies appears to have done (or at least they did not show or mention such validation).*

**If obscurin does not exert RhoGEF activity towards RhoA (etc), could the authors speculate what additional biological functions the ‘pseudo-GEF’ could perform (scaffolding, …)? An experiment that mutates any of the identified phosphorylation sites affect RhoA localization (T/D or S/D mutants vs. T/A or S/A mutants or wildtype) may bring some more insights.**

*Scaffolding is indeed a plausible hypothesis and is a function which has also been reported for other pseudo-enzymes. We now mention this possibility in our discussion but leave further experimental exploration of this question to future work.*

**The coverage of the protein using mass-spec seems to be very low (Figure S13), suggesting that the authors may miss out on phosphorylation sites in peptides that were not detected by this method. Did the authors try an alternative method (e.g. phos-tag gel analysis of in vitro-phosphorylated DH-PH fragments, or usage of a Thr5798Ala mutant) to see if all phosphorylation by MST is abrogated or if there are additional phosphorylation sites present in the fragment?**

*We did not use Thr5798Ala mutations or other techniques for validation beyond what is shown in the manuscript. However, we would like to emphasize that mass-spectrometry was done after phosphopeptide enrichment, not on total digested peptides. Thus, low coverage of protein regions without phosphosites is to be expected.*

**How did the authors verify that the domains are properly folded? Did the authors e.g. perform CD spectrum analysis to measure secondary structure and compare with expected 2nd structure content based on bioinformatics analyses?**

*Folding was evaluated via the characteristics of the 1D NMR spectra and relied on information such as peak dispersal up to >9 ppm and peaks below 0 ppm, indicating the presence of a hydrophobic core. See e.g.* [*http://www.pnas.org/content/102/6/1901*](http://www.pnas.org/content/102/6/1901) *for other examples of this approach. Further secondary structure analysis was not performed.*

**Reviewer #2: (…)
Comments:**

**1) I have a question about data discussion: why the authors seem to ignore that the entire SH3-DH-PH domain has never been tested in their experiment? The authors themselves introduce the RhoGEF domain as a “ SH3-DH-PH domain triplet”. Indeed, the requirement of the entire domain may represent a pre-requisite for proper functioning of the RhoGEF domain. This should be discussed.**

*We thank the reviewer for pointing this out. We now include experiments with the SH3-DH-PH both in absence and presence of the physiological PH-domain ligand P(3,4)IP_2_.*

**2) In light of these unexpected results, validating the in-vitro approach with in-vivo experiments becomes crucial. In figure S14 the authors tried to obtain such validation. In the system used in this work (neonatal cardiomyocytes), endogenous RhoA does not show a recognizable association with any particular region of the sarcomere as opposed to what was previously shown in skeletal muscle models. In addition, exogenous expression of the GFP-tagged DH-PH domain results in the formation of what might seem (at least judging by the reported images) as protein aggregates, possibly suggestive of protein misfolding, also not clearly associated with a particular region of the sarcomere. TC10 shows a more defined distribution, but the problem of the expression and localization of GFP-DH-PH persists. For the reasons above, I do not think that figure S14 provides valuable information as it is now. I would suggest using a different system (e.g. derived from skeletal muscle) to judge the potential rearrangement of Rho and TC10 in the presence of the Rho-GEF domain and possibly testing different GFP-tagged Rho-GEFs construct to evaluate and improve the quality of their exogenous expression and localization.**

*We thank the reviewer for this remark and agree that optimizing the experimental system and expression construct could be helpful. Unfortunately, due to the mentioned practical limitations (see our general response above), we are currently unable to conduct further cell-biological experiments for this project anymore. However, we want to emphasize that we always attempted to provide a careful interpretation of our results, see e.g.* line 475ff:

*“However, we cannot rule out the possibility that obscurin might activate RhoA or RhoQ/TC10 without changing their localisation, or that under different culture conditions or by using alternative cell types a difference would be revealed, warranting further work to test these possibilities.”*

*We also stressed the need for future cell biological / in vivo experiments in both the abstract and discussion. Additionally, if the journal permits, we are happy to publish the reviewer comments alongside the article so that others in the field can consider the reviewers’ suggestion in their experimental design.*

**3) The phosphorylation by CAMKII of the SH3 domain but not of the SH3-DH domain is possibly indicative of intramolecular interactions between the SH3 domain and the downhill DH. Such interactions might be important for the catalytic activity of the DH domain. The authors created different constructs, including a couple (Mermaid II and a zebrafish construct) containing the entire SH3-DH-PH fragment. Those constructs, though, were not tested for their catalytic activity. Is there a reason? Would it be possible to see data about the catalytic activity of these fragments on different GTPases to rule out a possible role of the SH3 domain in the catalytic activity of these isolated fragments?**

*We thank the reviewer for pointing this out. While the purification of mermaid II was not successful, we now included in vitro experiments with zebrafish SH3-DH-PH and RhoA and RhoQ/TC10.*

**4) In the aligned structures reported in figure 1, it appears that a noticeable difference between Dbs and Obscurin is the presence of Histidine 5879 in very close proximity to the switch region of RhoA and Cdc42. Have the authors considered if such a difference could lead to steric hindrance for the interactions with RhoA and/or cause reduced flexibility of the switch domain with potential consequences on the catalytic activity?**

*The obscurin structure in this figure is a homology model used to assess the hypothesis that the three mentioned residues could be functionally important and to guide further construct design and for this we were content with the finding that they are roughly in a similar position. Being a homology model, however, we are unsure whether conformation and position and orientation of the side chains are predicted with high enough accuracy to allow such interpretation. We have carried out additional molecular dynamics simulations that do indeed suggest that a significant amount of conformations may clash with RhoA binding. However, the nucleotide exchange reaction is a dynamic multistep process involving several structural changes (binding of Rho:GXP to the GEF and remodeling of the nucleotide binding pocket, release of GXP, rebinding of GXP to the Rho/GEF complex, dissociation of Rho:GXP from the GEF) and the Dbs:Rho structure only provides a static snapshot of a single step, so we are cautious not to overinterpret this data.*

**minor concerns:
1) Reference 1 and 10 appear a bit dated compared to the concept expressed.**

*We added two more recent references.*

**2) Figure 1D is not acknowledged**

*We thank the reviewer for pointing this out and added a reference to Figure 1D.*

**3) page7, line216: the authors introduce the experiments on RhoA and RhoQ localization as also performed on adult murine cardiomyocytes. There’s no trace of such cells throughout the manuscript.**

*A similar point was raised by reviewer 1, please see our above response.*

**4) Why the zebrafish DH-PH domain was used in MST2 phosphorylation assay (Fig.4B), if the Thr5798 residue is not conserved in that species, and the Ser5669 seem to be not included in the zebrafish DH-PH construct (5872-6217, according to figure 4B) ? Was it used as experimental control?**

*We included the zebrafish DH-PH construct because at this stage we did not yet know the exact MST2 phosphoylation site (or sites) (the mass spec analysis was performed later). From figure 4A we concluded that the phosphorylation site is either in the SH3/DH-interdomain linker (e.g. Ser5669), or that it is in the DH domain but that part of the linker is necessary for kinase binding/activation. The included zebrafish fragment does not feature the equivalent to Ser5669 but much of this SH3/DH-interdomain linker. In addition, we considered it possible that MST might also phosphorylate other residues e.g. of the PH-domain.*

**5) The authors have tested 21 DH fragments and they choose two of them. Can the authors explain the reason for this choice?**

*Fragment 5667-5899 was chosen because it was largest one of the soluble DH constructs containing also parts of the adjacent interdomain linkers. Because such linkers can sometimes exhibit autoinhibitory activity on DH domains, we also chose a slightly smaller one with slightly fewer residues at both the N- and C-terminal end, Fragment 5681-5889. There was no particular reason for 5681-5889 as compared to the other smaller fragments. We added a clarification to the manuscript to explain the reasons for our choice (see line 318-319).*

**6) The authors find no nucleotide exchange activity of any of the DH or DH-PH fragments. Did the authors test higher fragment concentrations?**

*With the human DH fragments we did a single (n=1) experiment in which we successively added different constructs to RhoA:mGDP up to a cumulative concentration of 15 μM - without apparent effect. Based on our experience with other GEFs, we felt 10 μM is a sufficiently generous amount and that the time over which we measured the reaction should have been long enough to detect any significant increase in nucleotide exchange compared to intrinsic exchange.*

**7) RhoQ/TC10 was found to be activated by an obscurin fragment covering the C-terminal region from 5679 to 6620. Did the authors test a similar fragment as a positive control for their nucleotide exchange activity and binding experiments?**

*The largest human construct which we (unsuccessfully) attempted to purify was 5686-6201, covering the DH-PH domains and the following two C-terminal Ig-domains. We then decided for the systematic boundary optimisation approach described in Figure 1. The largest construct which we purified is the zebrafish SH3-DH-PH construct, which we used in the GEF assays. Since we are unaware of examples in which Ig-domains contribute to the activity of RhoGEFs, we did not see mechanistic necessity to test larger constructs.*

**Minor issues**

**The fonts used to label some figures are small and hard to read. This issue is especially noticeable in Fig1 B and Fig 3D. Please use bigger fonts.**

*Figure 1B is actually a scanned in graph from a printout (the raw data was lost due to a power cut so we could not generate a new plot). However, we manually replaced axis-labels and the numbers for the x-axis with bigger fonts. The important information from this plot is the shape of the peak, showing a homogenous protein with no obvious signs of degradation products, impurities or oligomeric species. We also increased scale and font of several other figures (including 3D) for improved readability.*

**Figure 2: The scale of the Y-axis in Rac1 + Control Graph is slightly different from all the others. It would be ideal to use the same scale for all graphs.**

*We now use the same scale for all graphs.*

**The location of Ser5669 is described ambiguously. In line 203, it is said to be part of the interdomain linker between SH3 and DH, while in figure S15 it is represented as part of the SH3 domain. Please correct the ambiguity.**

*We corrected the ambiguity in Figure S16 (formerly S15).*
